# Nanosponge hydrogel of octadecyl 3-(3,5-di-tert-butyl-4-hydroxyphenyl) propanoate of *Alcaligenes faecalis*

**DOI:** 10.1007/s00253-023-12819-3

**Published:** 2024-01-12

**Authors:** Sayed E. El-Sayed, Neveen A. Abdelaziz, Ghadir S. El-Housseiny, Khaled M. Aboshanab

**Affiliations:** 1 Department of Microbiology and Immunology, Faculty of Pharmacy, Ahram Canadian University, Sixth of October City, Giza, 12451 Egypt; 2 Department of Microbiology and Immunology, Faculty of Pharmacy, Organization of African Unity St, Ain Shams University, Abbassia, 11566 Cairo Egypt

**Keywords:** Antifungal, β-Cyclodextrin nanosponges, *Alcaligenes faecalis*, Di-tert-butylphenol, Optimization, Box-Behnken design (BBD), Hydrogel, In vivo studies

## Abstract

**Abstract:**

Octadecyl 3-(3,5-di-tert-butyl-4-hydroxyphenyl) propanoate (ODHP) was extracted in a previous study from the culture broth of soil isolate *Alcaligenes faecalis* MT332429 and showed a promising antimycotic activity. This study was aimed to formulate ODHP loaded β-cyclodextrins (CD) nanosponge (NS) hydrogel (HG) to control skin fungal ailments since nanosponges augment the retention of tested agents in the skin. Box-Behnken design was used to produce the optimized NS formulation, where entrapment efficiency percent (EE%), polydispersity index (PDI), and particle size (PS) were assigned as dependent parameters, while the independent process parameters were polyvinyl alcohol % (w/v %), polymer-linker ratio, homogenization time, and speed. The carbopol 940 hydrogel was then created by incorporating the nanosponges. The hydrogel fit Higuchi’s kinetic release model the best, according to in vitro drug release. Stability and photodegradation studies revealed that the NS-HG remained stable under tested conditions. The formulation also showed higher in vitro antifungal activity against *Candida albicans* compared to the control fluconazole. In vivo study showed that ODHP-NS-HG increased survival rates, wound contraction, and healing of wound gap and inhibited the inflammation process compared to the other control groups. The histopathological examinations and Masson’s trichrome staining showed improved healing and higher records of collagen deposition. Moreover, the permeability of ODHP-NS-HG was higher through rats’ skin by 1.5-folds compared to the control isoconazole 1%. Therefore, based on these results, NS-HG formulation is a potential carrier for enhanced and improved topical delivery of ODHP. Our study is a pioneering research on the development of a formulation for ODHP produced naturally from soil bacteria.

**Key points:**

• *Octadecyl 3-(3,5-di-tert-butyl-4-hydroxyphenyl) propanoate was successfully formulated as a nanosponge hydrogel and statistically optimized*.

• *The new formula exhibited in vitro good stability, drug release, and higher antifungal activity against C. albicans as compared to the fluconazole*.

• *Ex vivo showed enhanced skin permeability, and in vivo analysis showed high antifungal activity as evidenced by measurement of various biochemical parameters and histopathological examination*.

**Supplementary Information:**

The online version contains supplementary material available at 10.1007/s00253-023-12819-3.

## Introduction

Although fungal infections are mostly unknown to the public, their total annual mortality is comparable to or exceeds that of malaria, tuberculosis, or HIV. The impact of fungal infections has been worsened by the steady increase of antifungal drug-resistant strains and species, which indicates the widespread consumption of antifungals for preventive measures and treatment and has been related, in the case of azole resistance in *Aspergillus*, to antifungal use in agriculture (Gow et al. 2022). The COVID-19 pandemic exacerbated the present situation by predisposing patients in intensive care units (ICUs) to secondary deadly fungal infections, making accurate diagnosis more challenging (Li and Denning 2023). Mucormycosis, also known as “Black Fungus,” has been linked to COVID-19 patients, making up approximately 70% of all mucormycosis cases reported earlier (Dam et al. 2023). When the pre- and post-COVID-19 eras are compared, there is an increased rate of *Candida albicans* coinfections during COVID-19 with impaired immune response to such infections (Moser et al. 2021; Zand et al. 2023). Furthermore, *Aspergillus fumigatus* has been identified as the most common cause of fungal infections in severely ill COVID-19 patients (Szabo et al. 2021).

For the treatment of medical cases and superficial skin infections, dermatologists favor topical semisolid formulations which provide effective delivery of drugs to the preparation methods and vehicles used, which have an impact on the pace at which the drugs permeate the skin (Akhtar et al. 2015). Additionally, they have a number of advantages over parenteral or peroral dosage forms, such as enhanced patient compliance due to their simplicity of use and noninvasive design, on-site delivery that minimizes systemic side effects, and efficient targeting properties (Wong et al. 2023). Out of the topical dosage forms, hydrogels are getting popularity due to their swelling ability in conjunction with their adhesion strength and potential to regulate drug release (Dattilo et al. 2023). The use of polysaccharide biopolymers in medicine is urgent because they are non-carcinogenic, non-allergenic, non-irritant, and non-toxic. β-Cyclodextrins (β-CD) are biodegradable, non-toxic materials and are gaining popularity because of their capacity to bind non-covalently to medication molecules (Santos et al. 2023).

Nanosponges (NS) are an innovative formulation that consists of a sponge-like structure that is non-collapsible and porous and is used to encapsulate nanoparticles (Das et al. 2023). It is mainly used in the pharmaceutical industry because it combines the benefits of nanoscale vesicular structures and microsponges making it ideal for cosmetic and cosmeceutical applications (Kandekar et al. 2023). The porous structure affects the release pattern in addition to allowing us to entrap a wide range of active molecules (Kandekar et al. 2023). Cyclodextrin nanosponges have excellent properties such as the simple method of preparation, the ability to form inclusion complexes, which incorporate less water-soluble molecules into their core cavities, improving drug aqueous solubility while also increasing bioavailability, biocompatibility, and stability and minimizing undesirable side effects (Lee and Poh 2023). When NS and hydrogel are combined together, this provides remarkable benefits due to its three-dimensional porous structure, the most significant of which is improved skin retention, improved patient compliance, decreased dose, and less adverse effects (Tiwari and Bhattacharya 2022).

To create an optimized product with superior attributes and quality, software-based optimization methods are used (Thirunavukkarasu et al. 2023). Response surface methodology (RSM) is an efficient statistical tool that uses lower-order polynomial equations to develop, improve, and optimize a process with many factors that influence the response (Chen et al. 2023). RSM reduces the overall number of possible combinations, saving time and materials during experimentation (El-Sayed et al. 2020b).

In our previous study, octadecyl 3-(3,5-di-tert-butyl-4-hydroxyphenyl) propanoate (ODHP) was extracted from *Alcaligenes faecalis* culture broth as an antifungal agent and was optimized for its maximum yield production (El-Sayed et al. 2020a). The toxic lipophilic phenol moiety 2,4-di-tert-butylphenol (DTBP) has been found in at least 169 species of organisms, including nitrogen-fixing cyanobacteria (Zhao et al. 2020), food-borne and hot spring Gram-positive bacteria (Aissaoui et al. 2019) and freshwater and soil Gram-negative bacteria (Dharni et al. 2014; Padmavathi et al. 2014), and fungi (11 species of eight families) (Zhao et al. 2020). Hence, our study was intended to optimally formulate ODHP into a nanosponge hydrogel and evaluate the effectiveness of the developed formulation in vitro and pre-clinically to be used as a potential formula for the treatment of topical mycotic infections.

## Materials and methods

### Chemicals, media, and antifungals used

β-Cyclodextrin (β-CD), diphenyl carbonate (DPC), carbopol 940, and poloxamer 188 were purchased from Middle East Company (Cairo, Egypt). Carboxymethyl cellulose (CMC), sodium alginate, hydroxylpropyl methylcellulose (HPMC E4), and Tween 80 were purchased from El-Nasr Pharmaceuticals (ADWIC, Cairo, Egypt). Triethanolamine (TEA), dichloromethane, methyl paraben, propylene glycol, and polyvinyl alcohol (PVA) were purchased from Sigma-Aldrich (St. Louis, MO, USA). Other materials used are as follows: Sabouraud Dextrose Agar (SDA) (Oxoid Ltd, England), collagenase 0.6 IU (Iruxol®, Abbott Co., and Wiesbaden, Germany); fluconazole (SEDICO Pharmaceutical Company, Giza, Egypt), and isoconazole (ISN) 1% (Candicure®, Al-Esraa Pharmaceutical Optima Co., Egypt).

### Extraction of ODHP

ODHP was optimized for its maximum yield production, extracted and purified from *Alcaligenes faecalis* culture broth in our lab as previously reported (El-Sayed et al. 2020a). The *Alcaligenes faecalis* MT332429 was previously identified using 16S ribosomal RNA (NCBI GenBank accession number MT332429) (https://www.ncbi.nlm.nih.gov/nuccore/MT332429) (accessed on 23 July 2023), and the isolate was deposited in the Culture Collection Ain Shams University (CCASU) belonging to the World Data Centre for Microorganisms (WDCM) under the code, *Alcaligenes faecalis* CCASU-MT332429 (http://ccinfo.wdcm. org/collection/by_id/1186) (accessed on 23 July 2023).

### Preparation of ODHP-loaded nanosponges (NS) by emulsion solvent diffusion method and optimization using RSM

Using the emulsion solvent diffusion approach, batches of nanosponges (NS) were formulated using varied ratios of β-cyclodextrin and diphenyl carbonate, as previously described (Sharma and Pathak 2011). In brief, two phases were adopted in this process, namely a continuous phase and a dispersed phase. The dispersed phase was prepared ultrasonically by stirring different molar ratios of β-cyclodextrin and cross-linking diphenyl carbonate with 105 µg ODHP in 20 mL dichloromethane for 10 min; this solution was prepared using a homogenizer (Ultra-Turrax, Germany). Then, 0.5% w/v PVA was dissolved in 150 mL of distilled water to make a continuous aqueous phase by stirring in a 60 °C water bath. The dispersed phase was slowly incorporated in the continuous phase using a syringe, while the reaction mixture was being emulsified at 35 °C using probe sonication (Fisher Scientific, Waltham, MA, USA) for 10 min and then left to homogenize. The dispersion produced (the nanosponges formed) was filtered using 0.45-µm filter paper to separate the solid mass and washed with deionized water and ethanol/methanol (50:50) in order to remove the free/un-reacted β-CD residues. It was then filtered once more, transferred to glass vials, and pre-frozen at − 80 °C for 12 h then lyophilized to remove any residual solvent (CHRIST, Osterode, Germany) using mannitol as cryoprotectant and under conditions of temperature equal to − 48 °C and pressure 0.37 mbar for 2 days to get the freely flowing powder (Fonte et al. 2016; Prabhu et al. 2021). The finished product was a fine white powder which was packaged and stored in airtight containers (Ghose et al. 2020). The Box-Behnken design was employed for the optimization process. Independent process parameters included β-cyclodextrin (polymer) and diphenyl carbonate (cross-linker) ratio, homogenization time (min), homogenization speed (rpm), and polyvinyl alcohol (PVA) % (w/v %). The optimization process dependent parameters were PDI, PS (nm), and entrapment efficiency% (EE) (Nait Bachir et al. 2019; Srivastava et al. 2021). The levels of independent parameters and goals of dependent ones are presented in Table 1. As shown in Table 2, the Box-Behnken design specified 29 experimental runs yielding quadratic equations that define the models.

**Table 1:** Independent and dependent variables of Box-Behnken design with their respective levels and goals

Independent variables	Levels of variables		
	− 1	0	1
A: polymer (β-CD)-cross-linker (diphenyl carbonate) molar ratio	1:2	1:4	1:10
B: homogenization speed (rpm)	10,000	12,500	15,000
C: homogenization time (min)	10	12	14
D: polyvinyl alcohol (%w/v)	0.3	0.4	0.5
Dependent variables	Target goal		
Particle size (nm)	Minimize		
Polydispersity index (PDI)	Minimize		
Entrapment efficiency %	Maximize

**Table 2:** Box-Behnken design runs for the four different factors tested showing the observed and predicted responses

Run	A: polymer-linker ratio	B: homogenization speed	C: homogenization time	D: PVA%	Particle size	Predicted particle size	PDI	Predicted PDI	EE	Predicted EE
1	1:10	12,500	14	0.4	363	359.79	0.5	0.46	86.96	87.37
2	1:4	12,500	12	0.4	320	324.41	0.21	0.14	89.67	89.38
3	1:4	12,500	10	0.5	326	326.24	0.27	0.28	89.86	89.57
4	1:4	10,000	10	0.4	333	335.46	0.31	0.31	87.21	87.62
5	1:4	15,000	12	0.3	355	346.46	0.44	0.40	88	88.08
6	1:4	10,000	12	0.5	342	336.12	0.33	0.34	87.85	88.16
7	1:10	12,500	10	0.4	359	360.96	0.48	0.47	86.84	87.02
8	1:2	12,500	12	0.3	382	376.96	0.58	0.58	90.87	91.21
9	1:4	12,500	14	0.3	323	322.58	0.22	0.25	89.15	89.2
10	1:4	12,500	12	0.4	320	324.41	0.21	0.14	89.67	89.38
11	1:4	12,500	12	0.4	320	324.41	0.21	0.14	89.67	89.38
12	1:4	12,500	12	0.4	320	324.41	0.21	0.14	89.67	89.38
13	1:10	15,000	12	0.4	373	383.67	0.54	0.61	86.61	86.25
14	1:10	10,000	12	0.4	370	370.84	0.54	0.54	86.32	85.61
15	1:4	12,500	12	0.4	320	324.41	0.21	0.14	89.67	89.38
16	1:2	12,500	10	0.4	379	378.79	0.56	0.56	91.12	91.4
17	1:4	10,000	14	0.4	330	334.29	0.29	0.29	87.61	87.97
18	0.5	15,000	12	0.4	391	401.5	0.66	0.70	90.45	90.63
19	1:4	15,000	10	0.4	351	348.29	0.41	0.38	88.14	88.26
20	1:4	15,000	14	0.4	349	347.12	0.38	0.37	88.88	88.62
21	1:2	12,500	12	0.5	385	379.46	0.61	0.60	92.1	91.94
22	1:4	12,500	14	0.5	324	325.08	0.24	0.27	90.02	89.93
23	1:2	10,000	12	0.4	389	388.67	0.63	0.62	90.34	89.99
24	1:4	10,000	12	0.3	335	333.62	0.35	0.34	87.44	87.43
25	1:4	15,000	12	0.5	357	348.96	0.46	0.43	88.55	88.8
26	1:2	12,500	14	0.4	377	377.62	0.55	0.54	91.7	91.75
27	1:10	12,500	12	0.3	365	359.12	0.51	0.50	86.57	86.83
28	1:4	12,500	10	0.3	325	323.74	0.24	0.27	89	88.84
29	1:10	12,500	12	0.5	366	361.62	0.52	0.51	87	87.56

### Characterization of the prepared ODHP-NS

#### Percent yield of ODHP-NS

By weighing the nanosponges and the original excipients utilized in their synthesis, the % yield of nanosponges was estimated as shown in Eq. (1) (Omar et al. 2020):

1 $$\%yield=\frac{\mathrm p\mathrm r\mathrm a\mathrm c\mathrm t\mathrm i\mathrm c\mathrm a\mathrm l\;\mathrm w\mathrm t.\mathrm o\mathrm f\;\mathrm N\mathrm S}{\left(\mathrm{wt}.\mathrm o\mathrm f\;\mathrm d\mathrm r\mathrm u\mathrm g)+(\mathrm{wt}.\mathrm o\mathrm f\;\mathrm\beta-\mathrm{CD}\right)+(\mathrm{wt}\;\mathrm{of}\;\mathrm{DPC})}\times100$$

#### Evaluation of particle size, PDI, and zeta potential (ZP)

The dynamic light scattering (DLS) technique was utilized to evaluate the average PS, PDI, and ZP of the formulated ODHP-loaded NSs using Malvern Nano ZS Zetasizer (Malvern Instruments Ltd., Worcestershire, UK). Prior to analysis, each sample was appropriately diluted with distilled water and ultrasonically processed for 3 min to break up agglomerates and dissociate adherents. At 25 ± 0.5 °C, samples were measured in triplicate, and the results were demonstrated as a mean value with a standard deviation (± SD) (Anwer et al. 2019).

#### Entrapment efficiency (%EE)

The %EE of ODHP-NS was evaluated by direct method through centrifuging the ODHP-NS in methanol at 10,000 rpm for 30 min (Centurion Scientific-K240R) to separate nanosponge particles. The content of free drug in the supernatant was measured by a UV spectrophotometer (Shimadzu, 1700, Japan) at *λ* max of ODHP (285 nm) (Sharma and Pathak 2011), and the concentration was determined from the standard curve of ODHP (Fig. S1) (Monica and Gautami 2014; Priyanka et al. 2018). All the measurements were made three times, and average results were recorded. The %EE and drug loading capacity % (DL%) of ODHP loaded NS were calculated using Eq. 2 (Penjuri et al. 2016) and Eq. 3 (Kumar et al. 2021), respectively, as follows:

2 $$\mathrm{\%EE}=\frac{\mathrm{Actual\;drug\;content\;in\;NS\;}}{\mathrm{Theoretical\;content }(\mathrm{content\;of\;ODHP\;initially\;added})}\times 100$$

3 $$\mathrm{\%DL}=\frac{\mathrm{Weight\;of\;ODHP\;in\;NS\;}}{\mathrm{weight\;of\;NS}}\times 100$$

#### Scanning electron microscopy (SEM) analysis

A scanning electron microscope (JEOL JSM-SEM, model: JSM6330 LV, Tokyo, Japan) was employed to show the surface morphology of the optimized ODHP-NS formula. The optimized ODHP-NS was placed carefully on the SEM stubs and covered with gold. At various magnifications, scanning was done, and areas were recorded and processed to determine the spherical 3D structure of the NS formed (Penjuri et al. 2016).

#### Fourier transform infrared (FTIR) spectroscopy analysis

FTIR was done to ensure that there would be no interaction between the ODHP and the polymer. FTIR spectrometer (Shimadzu, Japan) was utilized to analyze the FTIR spectra of pure ODHP and optimized ODHP-loaded NSs. Before being pressed into translucent film, the sample was suitably diluted with crystalline potassium bromide (1:10 w/w). A sample holder was used to mount the film, and several different frequencies of spectra were captured ranging from 4000 to 400 cm^−1^ using spectral manager software (Almutairy et al. 2021).

#### Differential scanning calorimetry

Differential scanning calorimetry of ODHP and optimized ODHP-loaded NS was performed using TA instruments, California (Discovery DSC25 series), equipped with a Refrigerated Cooling System 90 (RCS 90), TA Instruments, New Castle, USA, and TRIOS software. The DSC was graduated with indium for the enthalpy heat and melting point. A heating rate (10 °C/min) was used with 30–400 °C (temperature range), under nitrogen purge (350 mL/min). For reference, the standard aluminum empty pan was employed (Kumar et al. 2021).

#### Formulation of topical hydrogel containing optimized ODHP-NS formula

To incorporate the optimized ODHP-loaded NS into a hydrogel formula, carbopol 940, sodium alginate, CMC, poloxamer 188, and HPMC E4 were all explored as gel formers in different quantities (0.2, 0.5, 0.8, 1, 1.2, 1.5 w/v %) to select the best gel former (Ahmed et al. 2021). A specified amount of the gelling agent in grams was introduced in portions and dissolved in 100 mL of a 30:70 propylene glycol:distilled water mixture and homogeneously distributed using a magnetic stirrer at 600 rpm followed by a 15-min stagnation process to release trapped air. To achieve transparency and a pH of 6.7–6.9, 2 mL triethanolamine was added (Yang et al. 2016). Then, 1 g of methyl paraben was added as a preservative, and the gel was completed to 100 mL with distilled water. To obtain a smooth gel with no lumps, the dispersion was continually swirled on a magnetic stirrer at 600 rpm for around 6 h until formed, and it was allowed to stand overnight to accomplish complete hydration (Moglad et al. 2020). The hydrogel formulations were then stored at 5 °C in firmly closed screw-capped containers for further studies (Aldawsari et al. 2015).

### Evaluation of the selected prepared nanosponge-loaded hydrogels

#### Homogeneity, spreadability, extrudability, swelling study, pH determination, and viscosity measurement

All of the prepared nanosponge gels were visually inspected for homogeneity (Gangadharappa et al. 2017). The spreadability of the hydrogel was also evaluated. A glass plate (5 × 5 cm) was filled with 100 mg of the generated hydrogel. A glass plate of the same size was dropped upon the first one from a height of 5 cm. A steady weight (0.5 kg) was placed on the glass plate surface and held in place for 1 min. Following weight removal, the diameter of the spread circle in centimeter was measured (Ahmed et al. 2021; Moglad et al. 2020). To verify the capability of a hydrogel to flow from collapsible tubes after a constant weight (500 g) was applied for 10 s. For each formula, this examination was carried out in triplicate; extrudability was recorded in g/s (El-Gayar et al. 2022). The hydrogel’s swelling behavior was investigated by inserting 0.5 g of hydrogel in previously weighed perforated aluminum foil. The weight growth as a function of soaking duration in 10 mL of phosphate buffer 6.8 at 37 °C was assessed in the experiment. Prior to measurement, an aluminum foil was hung up for approximately 15 min to remove the excess swelling media. Measurements were made until the hydration degree approached equilibrium, which was reached when three successive measurements yielded the same weight. The samples were withdrawn from the beakers at varied time intervals (30 min, 60 min, and 120 min) and placed on a dry surface for some time before being reweighed and calculated according to Eq. (4)(Ambala and Vemula 2015):

4 $$\mathrm{Swelling\;index\;}\left({\text{SW}}\right)\mathrm{\%}=\frac{\mathrm{Wt }-{\text{Wo}}}{{\text{Wo}}} \times 100$$

where (SW) % denotes the equilibrium swelling percentage, *W*_t_ denotes the swollen gel weight after time *t*, and *W*_o_ is the weight of hydrogel before swelling at zero time.

The pH of the selected ODHP-loaded NS-based topical hydrogel was estimated by a standardized pH meter (Edge pH series, Hanna instruments) at a temperature of 25 °C.To investigate the rheology of the optimized ODHP-NS-HG formulation, Brookfield DV-III ultra-programmable rheometer (AMETEK Brookfield, East Lyme, CT, USA) was employed (Kumar et al. 2021).

#### Determination of drug content

In 100 mL phosphate buffer (pH 7.4), about 50 mg of the best formulated HG was dissolved and agitated for 2 h before bath sonication for 2 min. This strategy is aimed at maximizing drug solubility under mechanical shaking. The absorbance of the solution was calculated at 285 nm after filtration. The drug amount contained in the hydrogel was calculated using the standard curve (Fig S1) (Zakaria et al. 2016).

#### In vitro drug release studies

The release of ODHP from ODHP-NS-HG was performed using the USP dissolution apparatus II by the paddle technique. To mimic the human skin state, the paddles revolved at 50 rpm, and the temperature was fixed at 37 ± 0.5 °C (Ng et al. 2010; Singh et al. 2020). In dialysis bags, 10 mg of the optimized ODHP-NS-HG was added. The dialysis bags were linked with paddles and inserted in the release medium (phosphate buffer of physiological pH = 7.4) (Ghose et al. 2020). After 0, 1, 3, 5, 7, 9, 11, 13, 15, and 24 h time intervals, 3 mL samples were extracted and substituted with an equal volume of fresh phosphate buffer pH 7.4 solution. The samples were filtered and spectrophotometrically analyzed for drug concentration at *λ* 285 nm. The drug release mechanism from the porous nanosponge matrix and kinetics was calculated by fitting the data into various kinetic models. The mechanism of drug release from nanosponges was determined via the correlation coefficient (*R*^2^) value as previously reported (Gaber et al. 2023).

#### Photodegradation study

At room temperature (25 ± 2 °C), photodegradation experiments were carried out using a UVA lamp with a wavelength range of 254–365 nm. The optimized ODHP-NS-HG formulation was the system under investigation. An aliquot (40 mg) of test gels was equally placed around the bottom of a beaker and then irradiated for 4 h. Following the exposure interval, the beaker was withdrawn, its contents quantitatively transferred into a 20-mL calibrated flask, and sonication was performed for 15 min. The resultant sample was volume adjusted (20 mL), filtered (0.45-mm membrane filters), and quantified using a UV spectrophotometer. The degree of photodegradation was determined by comparing the ODHP peak regions from irradiated samples to those obtained from evaluating an equivalent amount of non-exposed formulations (previously tested for drug content). Each sample was evaluated three times, and the findings were demonstrated as mean value standard deviation (± SD) (Kumar et al. 2022).

#### Stability study

The chosen medicated NS-HG formula was kept in amber glass containers at both room temperature and refrigerated at 5 ± 3 °C for 3 months followed by examining the physical appearance, rheological behavior, pH, and drug content (Pushpalatha et al. 2019).

#### In vitro antifungal activity

In Sabouraud Dextrose Agar (SDA) plates previously seeded with 100 µL of 0.5 McFarland *C. albicans* ATCC10231, wells were filled with 100 µL ODHP-NS-HG. Placebo HG, positive (fluconazole 105 µg/mL), and negative (DMSO) controls were also included. Inhibition zones in different petri dishes were measured in triplicate (Agrawal et al. 2021).

#### Cytotoxicity assay

Vero cells (kidney epithelial cells of African green monkey (*Cercopithecus aethiops*)) were seeded in 96-well plates (Thermo Scientific™ PCR Plate, 96-well, Waltham, MA, USA) at a density of 5000 cells/well. After 1-day incubation, 10 µL of ODHP-NS-HG and FLC dilutions (7.8, 15.6, 31.25, 62.5, 125, 250, 500, and 1000 µg/mL) were added to each well for 24 h. The 3-(4,5-dimethylthiazol-2-yl)-2,5-diphenyl-2H-tetrazolium bromide (MTT) assay was done after 37 °C incubation in a humidified 5% CO_2_ environment (Espíndola et al. 2022). The IC_50_ was calculated as previously determined (Korbášová et al. 2022).

#### In vivo studies

All rates received the standard care according to the standard guidelines during all the stages of the experiment.

#### Animals

Forty adult male Wistar albino rats weighing 200–220 g were used. They were purchased from the animal house facility of Faculty of Pharmacy, Ahram Canadian University, Giza, Egypt. Prior to the start of the study, the animals were acclimatized for 15 days. Animals were handled and were housing in accordance with the Care and Use of Laboratory Animals recommendations and ARRIVE guidelines (https://arriveguidelines.org) (accessed on 20 March 2023). The whole study was reviewed and approved by the research ethical committee of the Faculty of Pharmacy, Ain Shams University, Egypt (Protocol approval number: ACUC-FP-ASU-RHDIRB2020110301-REC#39).

#### Experimental design and thermal injury model

A third-degree burn wound infection with *C. albicans* ATCC10231 (containing about 7.5 × 10^7^ CFU) was induced in rats as previously reported (Okur et al. 2020). Briefly, the rats were divided into eight groups (Groups A–H; 5 rats each) as follows:

Group (A): Unburned, uninfected, treated with ODHP-NS-HG (skin irritation test).

Group (B): Control, burned, infected, untreated.

Group (C): Control, burned, uninfected, untreated.

Group (D): Control, burned, infected, treated with vehicle (negative control hydrogel).

Group (E): Burned, infected, treated with ODHP-NS-HG.

Group (F): Positive control-1, burned, infected, treated with collagenase 0.6 IU (Iruxol®, Abbott Co., Wiesbaden, Germany).

Group (G): Positive control-2, burned, infected, treated with isoconazole hydrogel (ISN) 1% (Candicure®, Al-Esraa Pharmaceutical Optima Co., Egypt).

Group (H): Normal control group (intact, unburned, uninfected, untreated).

#### Survival rate study

The tested formulations (1 g of hydrogel) were first applied 2 h after the induced infection to the burned skin and continued twice daily for 14 days. Three days after infection, the survival rate of the animals was determined. Dead animals were removed and calculated when calculating mortality rates. The animals were euthanized by cervical dislocation after being anaesthetized intraperitoneally with a cocktail of 60 mg kg^−1^ ketamine and 10 mg kg^−1^ xylazine, and the dorsal skin at the site of the lesion was carefully removed and kept in 10% formalin solution for histological investigation (Abdellatif et al. 2021).

#### Wound size measurement

Progression of the wound was photographed, and according to the wound diameter at different days of injury, the wound contraction % was calculated by Eq. (5) (Abdellatif et al. 2021):

5 $$\mathrm{Wound\;contraction\;\%}=\frac{\mathrm{Wt }-{\text{Wo}}}{{\text{Wo}}} \times 100$$

where *W*_t_ denotes the measured wound area at a given time interval and *W*_o_ is the initial wound area at the onset of the experiment (Tang et al. 2021).

#### Skin irritation studies

Dermal responses such as erythema and edema were reported for animals in group A (unburned, uninfected treated with 1 g ODHP-NS-HG) based on a visual scoring scale as previously described (Wairkar et al. 2022).

#### Histopathological examination

As previously reported, all conventional methods for sample fixation and staining were adhered to in accordance with standard practices (Al-Sabaawy et al. 2021). The Masson’s trichrome staining technique was employed to assess the quantity of collagen fibers within the tissues and perform an unbiased histological analysis. In accordance with established methods, six distinct and non-overlapping sections from the dermal layers of each sample were randomly selected and subjected to scanning in order to quantify the proportionate area occupied by collagen fibers (Negm et al. 2022).

#### Ex vivo skin permeation analysis

The investigation of skin permeation involved the use of normal abdominal albino rat skin, and the assessment was conducted using the Franz diffusion cells with a receptor volume of 22.5 mL and a diffusion area of 3.104 cm (Hussain et al. 2016). The positive control ISN1% and 100 mg of the formulation were both evenly applied to the skin. Throughout the experiment, the lower receptor chamber, which contained phosphate buffer (pH 7.4) and 2% v/v DMSO, was mixed continuously using a magnetic bead to approximate skin temperature. Samples were collected at predetermined intervals of 0.5, 1, 2, 4, 6, 8, 10, 12, 16, and 24 h and replaced with the same buffer media. The samples were filtered, and the contents of each were identified by spectrophotometric analysis at 285 nm for ODHP-NS-HG and 279.8 nm for ISN1%. Any residual formulation present on the skin surface was washed with PBS (pH 7.4) to assess drug penetration into rat skin (Hussain et al. 2016; Khurana et al. 2013).

#### Collection of blood samples

Blood samples were taken from retro-orbital plexus under lidocaine (4%) local anesthesia from each animal in all groups 3 days after the beginning of the study and before the rats were euthanized (Moustafa et al. 2018). The serum was utilized to measure levels of some immunological markers such as pro-inflammatory cytokines (tumor necrosis factor alpha (TNF-α), nuclear factor-kappa (NF-κB)-p105, interleukin-6 (IL-6) and interleukin-1 beta (IL-1β)) and the inflammatory marker cyclooxygenase (COX-2) prostaglandin-endoperoxide synthase (PTGS-2) and to state the level of angiogenesis by vascular endothelial growth factor (VEGF) (Nonoguchi et al. 2022).

#### Angiogenesis, inflammatory markers, and pro-inflammatory cytokine measurements

ELISA kit(s) based on the sandwich ELISA principle (Elabscience®) were utilized to assess inflammatory markers and pro-inflammatory cytokines. These kits include pre-coated micro-ELISA plates with antibodies specific to rat COX-2, TNF-α, VEGF, NF-κB-p105, IL-6, and IL-1. The relevant antibody was mixed with the samples (or standards) before being added to the micro-ELISA plate wells. Following that, an avidin-horseradish peroxidase (HRP) combination and a biotinylated detection antibody specific for the observed parameter were added successively to each microplate well and incubated at 37 °C for 30 min. After adding the substrate, wells containing the measured parameter, the biotinylated detection antibody, and the avidin-HRP conjugate will turn blue. The optical density (OD) was calculated spectrophotometrically at 450 nm ± 2 nm using a plate reader (BMG Labtech, FLUOstar Omega, Germany). For rats, the OD value is inversely proportional to the concentration of the parameter being measured (Alvarez et al. 2020; Navaei-Alipour et al. 2021).

### Statistical analysis

For the calculation of *p*-values and standard deviation, a one-way ANOVA test was applied followed by Tukey’s multiple comparison test (GraphPad Software Inc., San Diego, CA, USA). The results were presented as average values ± standard deviation. Data analysis, response surface generation, and model diagnostic plots were accomplished using Design-Expert® v. 11.0. The experimental data were statistically validated through analysis of variance (ANOVA), which determined the significance of each parameter.

## Results

### Optimization of ODHP-loaded nanosponges (NS)

The actual values of the investigated factors, design, observed versus the predicted results are displayed in Table 2. The resulting quadratic BBD model equations obtained from the software are as follows:

$$\mathrm{Particle\;size}=800.44934-628.6289\times A-0.064956\times B-0.291667\times C+12.50000\times D+1122.01705\times {A}^{2}+2.70091{\text{E}}-06\times {B}^{2}$$

$${\left(\mathrm{Polydispersity\;index}\right)}^{2}=2.70996-3.40867\times A-0.000331\times B+0.056240\times C-2.63667\times D+0.000019\times A\times B-0.019188\times A\times C+0.317500\times A\times D-5.85000E-07\times B\times C+0.000032\times B\times D-0.007625\times C\times D+5.84104\times {A}^{2}+1.32587{\text{E}}-08\times {B}^{2}-0.001761\times {C}^{2}+2.87542\times {D}^{2}$$

$$\mathrm{Entrapment\;efficiency \%}=50.26539+1095000\times A+0.005201\times B+0.089583\times C+3.62500\times D-2.02886E-07{B}^{2}$$

Table 3 shows the ANOVA results for the different responses, the *F*-values were 90.03, 84.26, and 123.90 (*p*-value < 0.0001) for PS, PDI, and EE%, respectively, which proves the models are significant. The model terms (*A*, *B*, *A*^2^, *B*^2^, *D*^2^) for PS, (*A*, *B*, *A*^2^, *B*^2^, *C*^2^, *D*^2^) for PDI, and (*A*, *B*, *D*, *B*^2^) for EE% all having *p-*values less than 0.05 are significant model terms. Also, a low coefficient of variation of 1.56, 10.33, and 0.3838 for PS, PDI, and EE%, respectively, was obtained, indicating the good reliability of the experimental values. The coefficients of determination *R*^2^ values which were 0.9609, 0.988, and 0.9642 suggested that 96.09%, 98.83%, and 96.42% of variability in PS, PDI, and EE% responses, respectively. The predicted *R*^2^ of 0.9275, 0.9324, and 0.9413 were in agreement with the adjusted *R*^2^ of 0.9502, 0.9765, and 0.9564. Finally, the signal-to-noise ratio(s), or adequate precision, were equal to 29.352, 29.391, and 40.816 for PS, PDI, and EE%, respectively.

**Table 3:** ANOVA table for particle size (PS) model, polydispersity index (PDI) model, and entrapment efficiency (EE%)

Source	Sum of squares	df	Mean square	*F*-value	*p*-value
Particle size	16,181.93	6	2696.99	90.03	< 0.0001
A-polymer-linker ratio	954.08	1	954.08	31.85	< 0.0001
B-homogenization speed	494.08	1	494.08	16.49	0.0005
C-homogenization time	4.08	1	4.08	0.1363	0.7155
D-PVA%	18.75	1	18.75	0.6259	0.4373
*A*^2^	13,902.45	1	13,902.45	464.09	< 0.0001
*B*^2^	1966.76	1	1966.76	65.65	< 0.0001
Residual	659.03	22	29.96		
Lack of fit	659.03	18	36.61		
Pure error	0	4	0		
Cor total	16,840.97	28			
Polydispersity index	0.4263	14	0.0304	84.26	< 0.0001
A-polymer-linker ratio	0.0264	1	0.0264	73.09	< 0.0001
B-homogenization speed	0.0099	1	0.0099	27.42	0.0001
C-homogenization time	0.0002	1	0.0002	0.6164	0.4455
D-PVA%	0.0005	1	0.0005	1.29	0.2745
AB	0.0004	1	0.0004	1.04	0.326
AC	0.0002	1	0.0002	0.652	0.4329
AD	0.0002	1	0.0002	0.4463	0.515
BC	0	1	0	0.0947	0.7628
BD	0.0002	1	0.0002	0.6908	0.4198
CD	9.30E − 06	1	9.30E − 06	0.0257	0.8748
*A*^2^	0.3541	1	0.3541	979.82	< 0.0001
*B*^2^	0.0445	1	0.0445	123.26	< 0.0001
*C*^2^	0.0003	1	0.0003	0.8911	0.3612
*D*^2^	0.0054	1	0.0054	14.84	0.0018
Residual	0.0051	14	0.0004		
Lack of fit	0.0051	10	0.0005		
Pure error	0	4	0		
Cor total	0.4313	28			
Entrapment efficiency	72.07	5	14.41	123.9	< 0.0001
A-polymer-linker ratio	57.55	1	57.55	494.74	< 0.0001
B-homogenization speed	1.24	1	1.24	10.67	0.0034
C-homogenization time	0.3852	1	0.3852	3.31	0.0818
D-PVA%	1.58	1	1.58	13.56	0.0012
*B*^2^	11.31	1	11.31	97.23	< 0.0001
Residual	2.68	23	0.1163		
Lack of fit	2.68	19	0.1408		
Pure error	0	4	0		
Cor total	74.74	28			

**Fig. 1:**
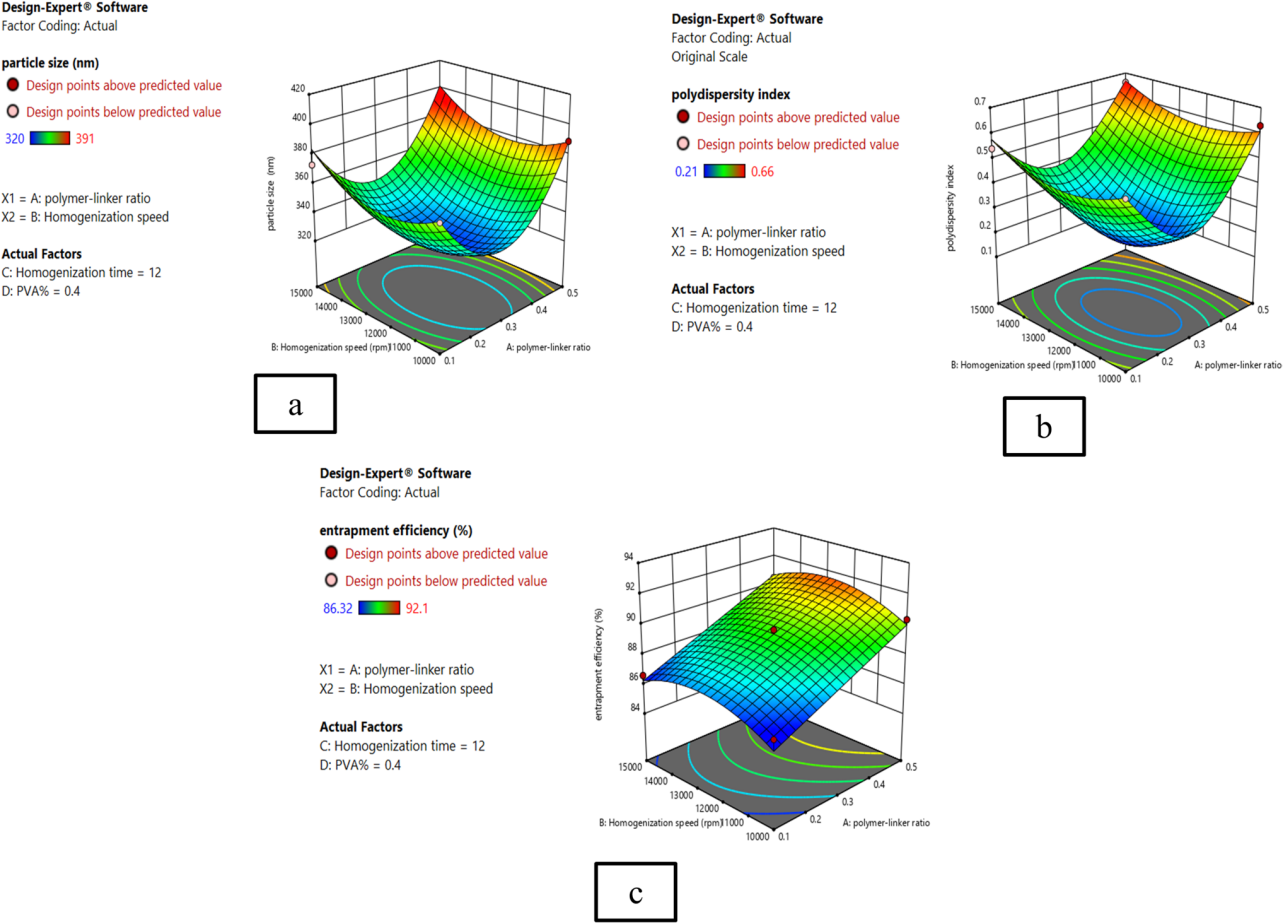
Three-dimensional response surfaces representing the effect of the four significant parameters on nanosponges’ dependent variables. **a** Particle size plot. **b** Polydispersity index plot. **c** Entrapment efficiency plot

Figure 1 shows the three-dimensional response surface plots (3D plots) which revealed that the optimum conditions for minimum particle size, minimum PDI, and maximum EE% were polymer linker molar ratio of 1:3, homogenization time 14 min, homogenization speed 12,460 rpm, and PVA% 0.5.

The normal probability plots of residuals for PS, PDI, and EE% (Fig. S2 a, b, and c, respectively) suggested that the residuals follow a normal plot as showed by the linear pattern.

The Box Cox plots are useful tools that determine the utmost applicable power transformation. As shown by our results, the current lambda (*λ* = 1) was adequate for PS and EE% models (Fig. S3 a and c, respectively). Polydispersity index model was transformed to the power 2 as suggested by the software (Fig. S3b).

The predicted versus actual values plot of PS, PDI, and EE% displayed an acceptable agreement between the projected and the actual data (Fig. S4 a, b, and c, respectively).

The residuals versus run number plot of PS, PDI, and EE% showed that the points were arbitrarily scattered around zero (Fig. S5 a, b and c, respectively) which point out that the model fits the data.

### Characterization of the prepared ODHP-NS

**Fig. 2:**
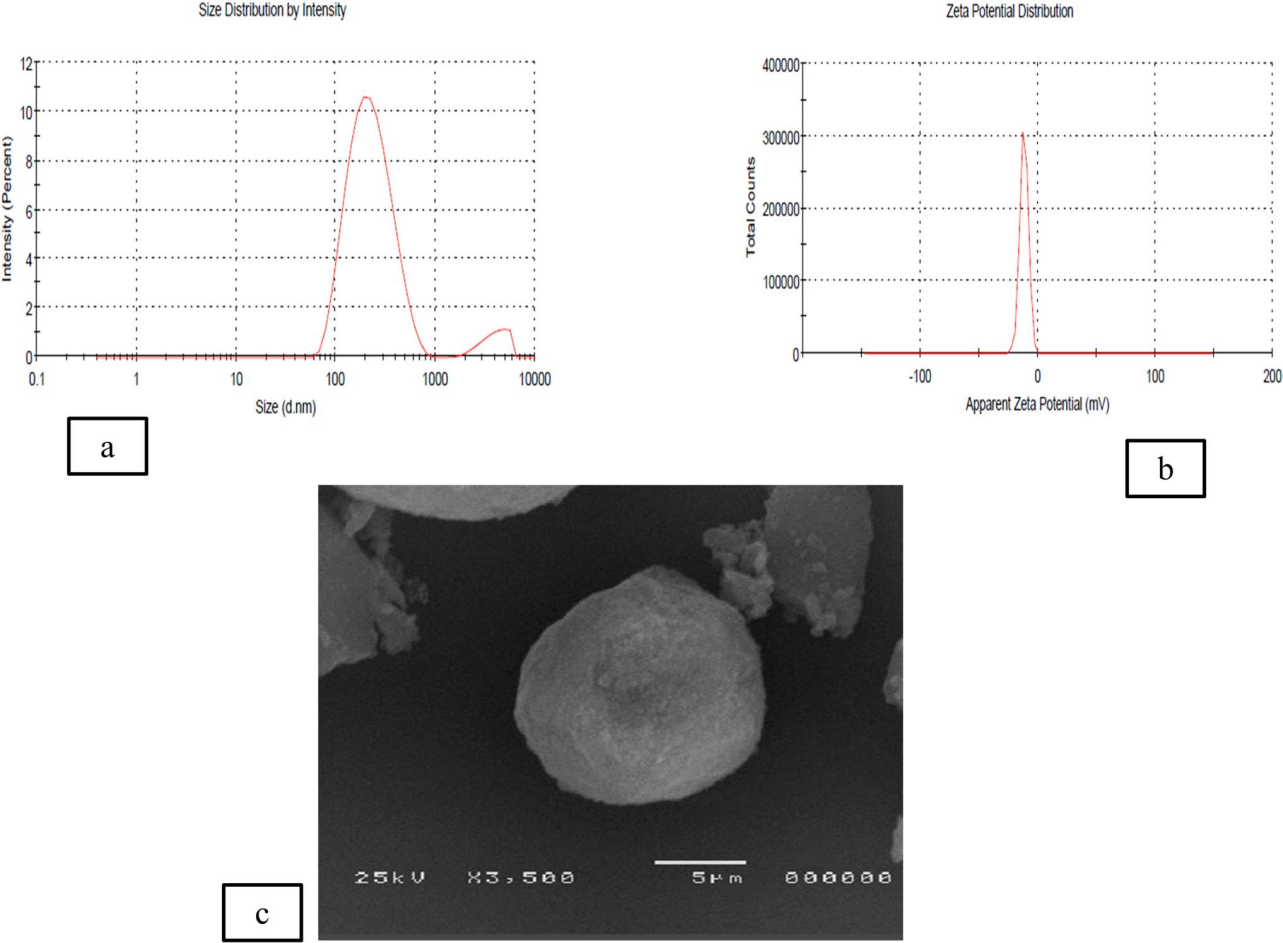
Particle size distribution by intensity (**a**), zeta potential distribution (**b**), and SEM image of the optimized ODHP-NS (**c**)

The mean %yield of ODHP-NS was found to be 84.4% ± 0.42. The PS of optimized ODHP-NS was 332 ± 1.35 nm, while PDI was 0.233 ± 0.02 Fig. 2a. The zeta potential was − 13.8 ± 0.56 mV. Particle size spreading by intensity for optimized ODHP-NS and zeta potential distribution are represented in Fig. 2b. Entrapment efficiency of optimized ODHP-NS was found to be 90.14 ± 0.65%, and drug loading % (DL%) was found to be 87.5 ± 0.77%. The SEM image of the optimized ODHP-NS is shown in Fig. 2c, which revealed a spongy nanosized spherical NS with a porous surface. The texture of the nanosponge makes it easier for the drug to penetrate its interpenetrating network.

### Results of FTIR spectroscopy analysis

Figure S6a shows the captured FTIR spectrum of the complex, which was very close to the FTIR spectrum of β-CD (Fig. S6b). This is because the complex and the host molecule have comparable chemical bonding properties. Furthermore, a broad hydroxyl band of β-CD at 3370.72 cm^−1^ was found to be narrowed in the inclusion complex FTIR spectrum. The frequencies for β-CD were found at 1157.84 cm^−1^, 1029.24 cm^−1^, and 2928.53 cm^−1^, respectively, which correspond to the symmetric and non-symmetric stretching of [CH2], [C–C], and bending vibration of [O–H]. The characteristic peaks of ODHP (Fig.S6c) disappeared in inclusion complex which suggests that these groups of the drug are included in the cavity.

### Differential scanning calorimetry

DSC thermograms of pure ODHP and optimized nanosponge formulation are presented in Fig. S7 a and b, respectively. The endothermic DSC curve of a ODHP was reflected as a sharp peak at 247.45 °C, corresponding to its melting temperature. DSC curve of optimized ODHP-NS exhibits a broad endothermic peak, formed by coalescence of β-CD and ODHP as a result of successful drug encapsulation in porous cavities of NS. The disappearance of sharp drug peak and upward line higher than the baseline (exothermic) curve represents fusion of drug with homogenous drug distribution in the polymer, signifying stable optimized ODHP-loaded NS.

### Formulation and evaluation of topical hydrogel containing optimized ODHP-NS formula

Carbopol 940 was the best gelling agent chosen for the ODHP-NS-HG preparation (Table S1). Different gel formulae were prepared using carbopol 940 with compositions ranging from 0.2 to 1.5% w/w (Table S2). However, the compositions of other constituents remained constant at a fixed concentration. The formula HG-5 was the best preparation and hence was selected for further studies. Table S3 demonstrates the results obtained for homogeneity and physical appearance of different carbopol 940-HG preparations.

The characterization parameters (spreadability, extrudability, equilibrium swelling study, pH, and viscosity measurement) for the selected hydrogel were determined, and the results were recorded. HG-5 exhibited a translucent appearance with a smooth and uniform consistency, showing the most optimal measured parameters. The spreadability was 5.74 ± 0.032 cm, while pH was 6.75 ± 0.13 confirming skin compatibility. The swelling index was denoted as 350.6 ± 0. 66%, while the extrudability was recorded as 0.97 ± 0.11 (g/s). Moreover, the viscosity was estimated to be 1092 ± 4 cps. The percentage of drug content for the selected HG-5 was 90.96 ± 0.32%, suggesting the drug was equivalently scattered throughout the gel.

### In vitro drug release studies

The release was best fitted into Higuchi diffusion kinetic model (coefficient of correlation *R*^2^ value = 0.9976). To study the mechanism of release of ODHP from the prepared formula, Korsmeyer-Peppas model was employed. A diffusion exponent (*n* values 0.563) indicated a non-Fickian model (anomalous transport) indicating that the drug release is monitored by the diffusion and polymer chain relaxation. The various kinetic models of the in vitro release data are represented in Fig. S8 a, b, c, d and e, respectively. A comparison between the correlation coefficient of the different used kinetic models is shown in Table S4.

### Photodegradation and stability studies

The drug content of the irradiated sample was 89.04 ± 0.51% with non-significant difference (*p* > 0.05) compared to non-exposed samples. After 3 months of storage in room temperature and in a refrigerator, no significant change could be noticed in the physical look, viscosity, pH, and drug content (*p* > 0.05) (Table S5).

### In vitro antifungal activity

ODHP-NS-HG showed enhanced in vitro antifungal activity against *C. albicans* ATCC10231 by 1.5-fold (35.6 ± 0.5 mm) compared to the positive control fluconazole which showed an inhibition zone of 24.4 ± 0.2 mm (Fig. S9).

### Cytotoxicity assay

IC_50_ values, corresponding to 50% inhibition of the cell growth in vitro of the optimized ODHP-NS and fluconazole (as reference standard), were 325.6 µg/mL and 287.8 µg/mL, respectively.

### In vivo studies

#### Survival rate study

The survival rates of the tested rats for each of the studied groups showed their maximum levels in positive control groups F and G and H and treated groups A and E with 100% survival rates, while they showed the same records for control groups B and D (40%) and were relatively higher in control group C (60%).

#### Wound size measurement

**Fig. 3:**
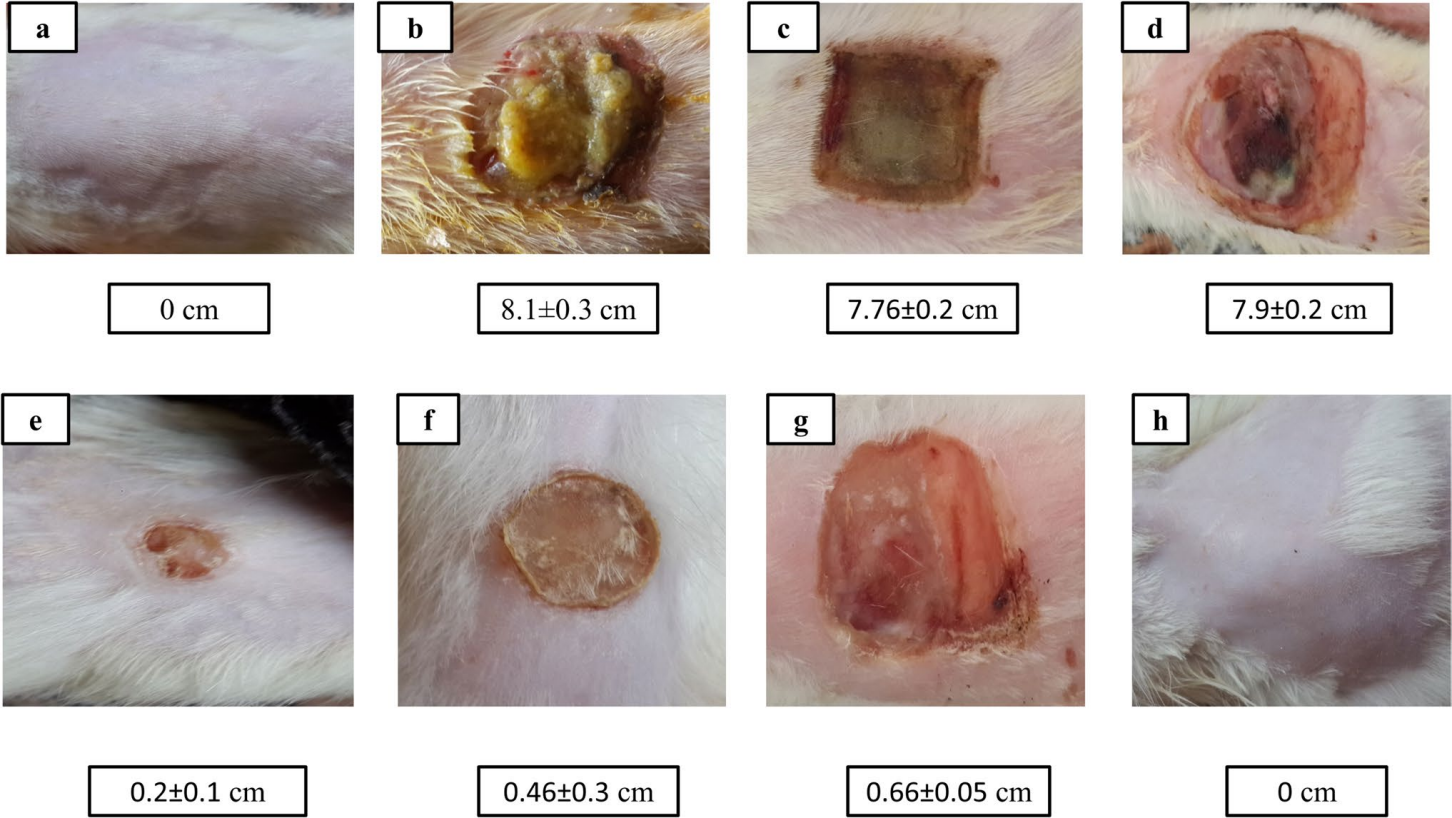
The photographs of the wounds of injured skin at 14th day of injury of a representative rat from each group with mean wound size (cm)

The wound size contraction on day 14 for group E was found to be 80%, which was 1.5-fold higher compared to positive control group F which showed only 54% contraction and 2.4-fold greater than positive control group G that showed 34% contraction. On the contrary, control groups B, C, and D showed impaired wound contraction as the wound size continued to enlarge. The mean wound diameters of group E were significantly different from those of groups B, C, D, and G (*p* < 0.05) and not significantly different from group F (*p* > 0.05). Wound photographs and data are presented as mean ± SD as shown in Fig. 3.

#### Skin irritation study

The applied ODHP-NS-HG formulation showed to be non-irritant and non-edematous on the rat intact skin. The final observations for erythema and edema scored zero.

#### Histopathological examination

**Fig. 4:**
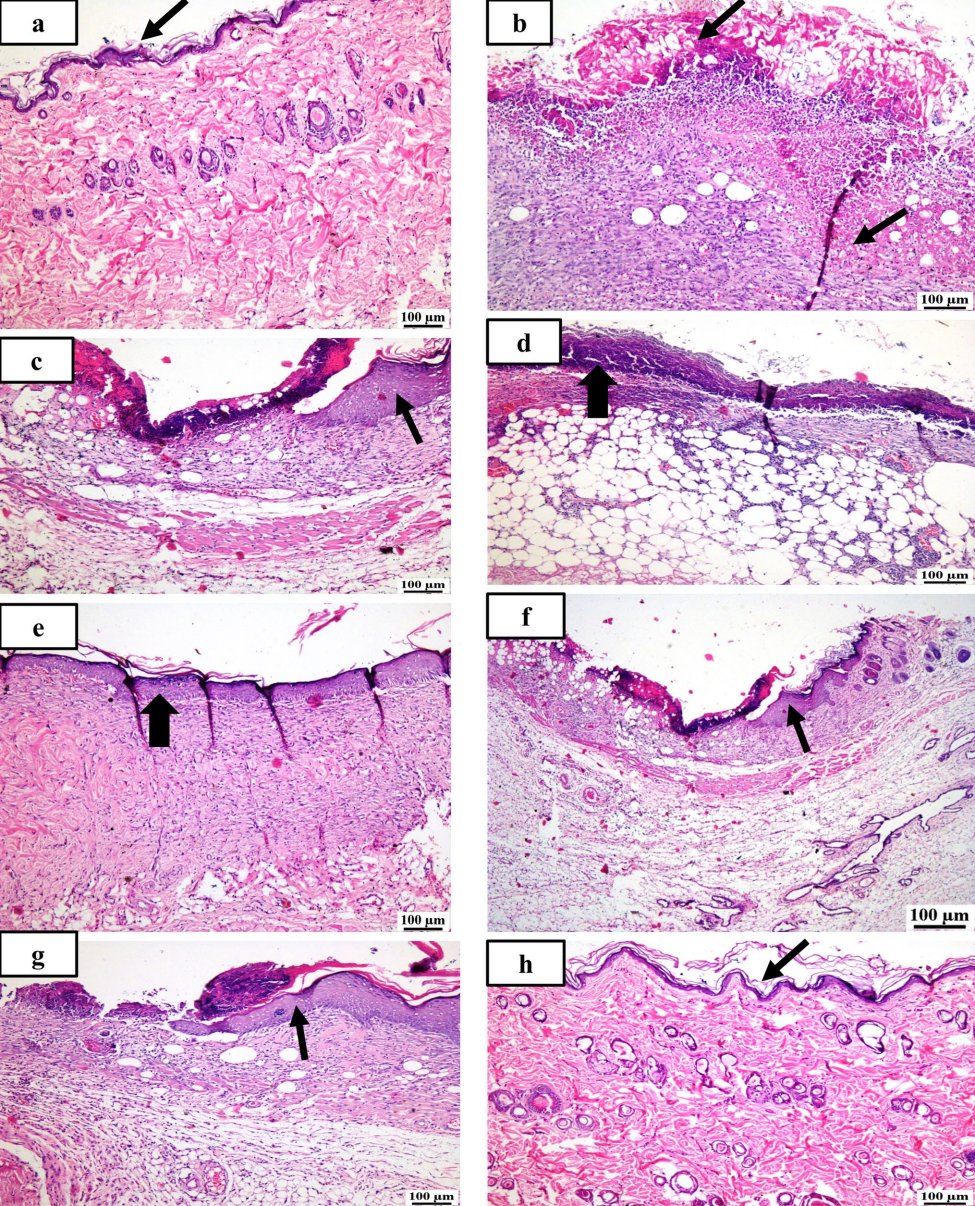
Photomicrographs of different skin layers and wound healing process stained with Hematoxylin and Eosin (H&E) of the different tested groups as follows: (**a**) unburned, uninfected, treated with ODHP -NS-HG (skin irritation test; (**b**) Control, burned, infected, untreated; (**c**) control, burned, uninfected, untreated; (**d**) control, burned, infected, treated with vehicle (negative control hydrogel); (**e**) burned, infected, treated with ODHP -NS-HG; (**f**) positive control-1, burned, infected, treated with Collagenase 0.6 IU (Iruxol®, Abbott Co., Wiesbaden, Germany); (**g**) positive control-2, burned, infected,  treated with Isoconazole hydrogel (ISN) 1% (Candicure®, Al-Esraa Pharmaceutical Optima Co., EGYPT); (**h**) normal control group (intact, unburned, uninfected, untreated). Arrows indicate significant and specific histopathological changes in the tissues of each group as indicated in the text

**Fig. 5:**
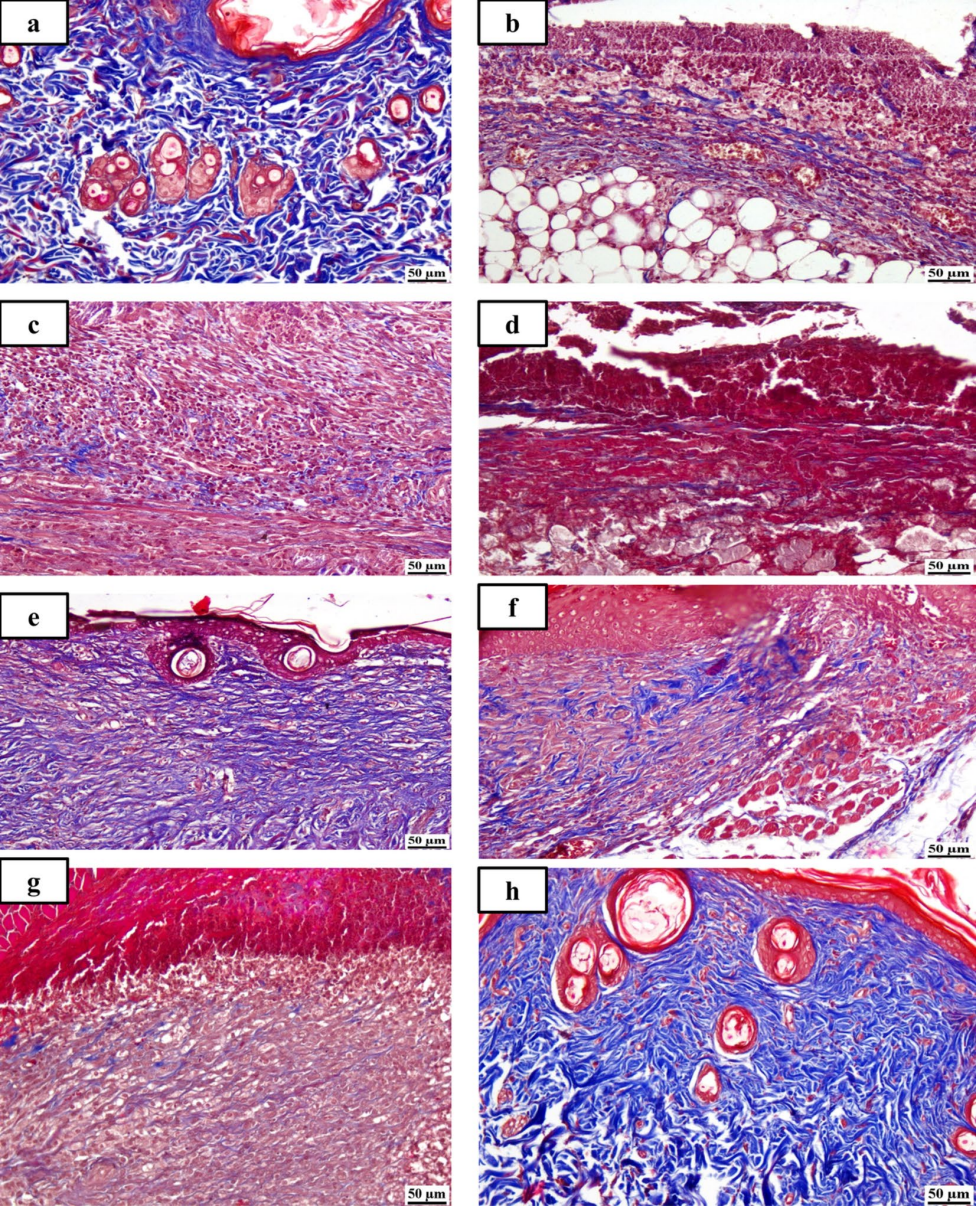
Photomicrographs of skin layers, wound healing, and collagen formation stained with for Masson’s trichrome staining (MTC) of the different tested groups as follows: (**a**) unburned, uninfected, treated with ODHP -NS-HG (skin irritation test; (**b**) Control, burned, infected, untreated; (**c**) control, burned, uninfected, untreated; (**d**) control, burned, infected, treated with vehicle (negative control hydrogel); (**e**) burned, infected, treated with ODHP -NS-HG; (**f**) positive control-1, burned, infected, treated with Collagenase 0.6 IU (Iruxol®, Abbott Co., Wiesbaden, Germany); (**g**) positive control-2, burned, infected,  treated with Isoconazole hydrogel (ISN) 1% (Candicure®, Al-Esraa Pharmaceutical Optima Co., EGYPT); (**h**) normal control group (intact, unburned, uninfected, untreated)

**Fig. 6:**
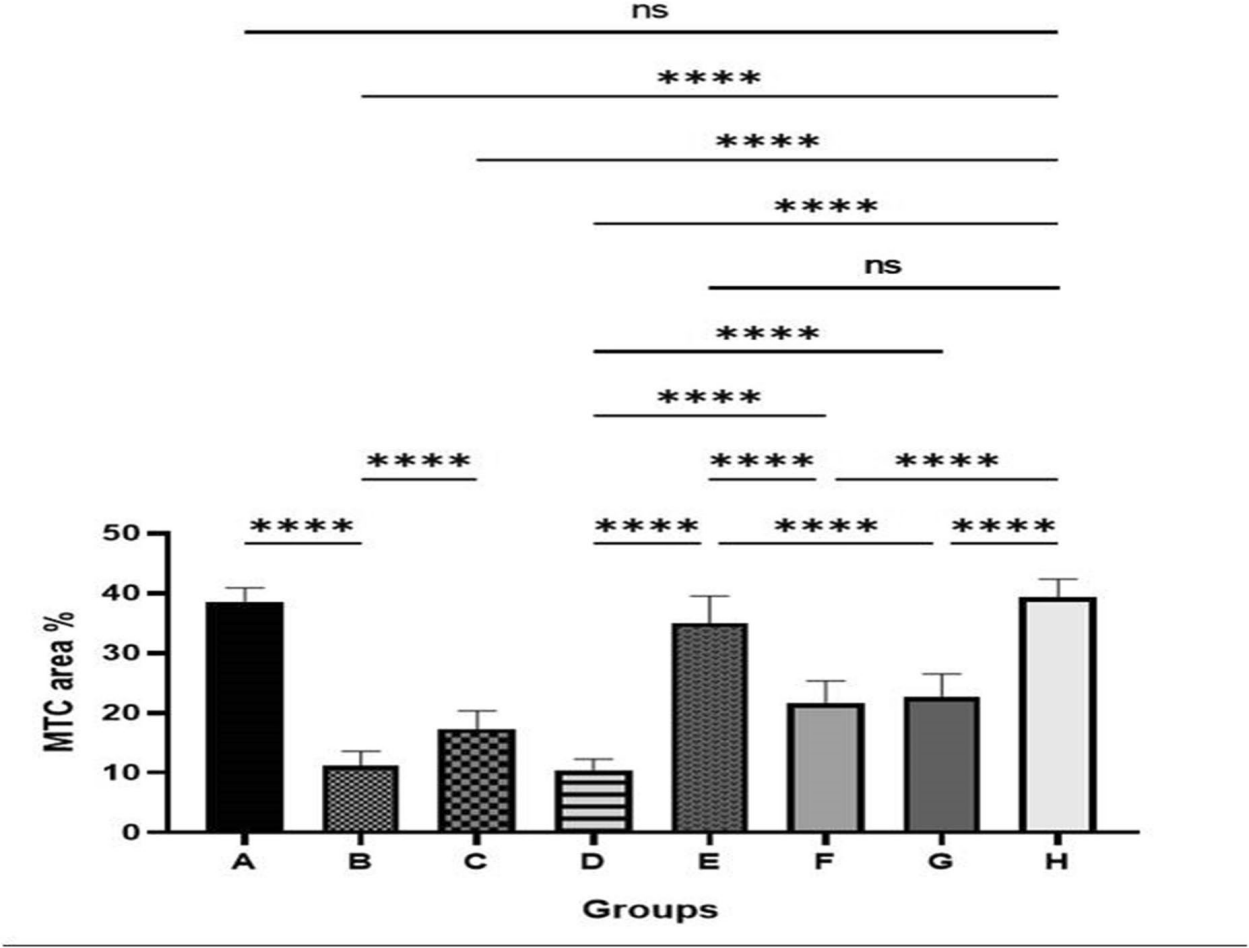
Bar chart showings area percentage of bluish stained sections in MTC staining for different groups. Data expressed as means ± SD. A significant difference is considered at *p* < 0.05

Photomicrographs demonstrating the histopathological features (hematoxylin and eosin (H&E)) of skin layers and wound healing process in different groups are shown (Figs. 4 and 5). Group (A): The typical histological structures of the various skin layers were visible in a skin photomicrograph, including an apparent intact dermal layer, integral epithelium covering an intact epidermal layer (Fig. 4a). Also, it demonstrated significant normally distributed collagen deposition, as seen in Masson’s trichrome–stained tissue segments of all the samples (MTC) (Fig. 5a), up to 38.6% of the mean area percentage of the dermal content (Fig. 6). Group (B) showed significant dermal collagen necrosis and an accumulation of necrotic tissue debris, highly inflamed granulation tissue fills the wound gap (arrows). Some localized dermal hemorrhagic patches and inflammatory cell infiltrates can be seen as well (Fig. 4b). Moreover, this group displayed less developed collagen fibers (Fig. 5b), up to 11.21% of the mean dermal layer content area (Fig. 6). Group (C) demonstrated moderate inflamed granulation tissues occupying the wound gap with necrotic crust covering and epidermal hyperplasia at the wound edge (arrows) (Fig. 4c). This group also displayed insignificant records of mature collagen (Fig. 5c) up to 17.31% of the mean area percentage of the dermal layer content ensuing lower collagen deposition in the wound gap (MTC) (Fig. 6). Group (D) exhibited similar findings of group B. Photomicrograph of skin showing intense inflamed granulation tissue filling the wound gap with severe necrosis of dermal collagen and accumulation of necrotic tissue debris (arrows) (Fig. 4d). Also, photomicrograph of skin showing limited collagen deposition in the wound gap (MTC) (Fig. 5d) up to 10.37% of the mean area percentage of the dermal layer content (Fig. 6). Group (E): Photomicrograph of skin showing higher and accelerated wound gap healing degree with full re-epithelization indicating hyperplastic epidermal remodeling and collagen rich filling granulation tissue. Also, Fig. 4e showed a higher fibroblastic activity and an expressive decrease in inflammatory cells (arrows) and displayed a higher records of dermal collagen fibers (Fig. 5e) up to 33.14% (almost threefold more when compared to group (B) (Fig. 6). Group (F): photomicrographs showed less inflamed granulation tissue and persistent records of a narrow ulcerated wound gap covered a necrotic tissue debris (Fig. 4f) with a moderate maturation in dermal collagen fibers (Fig. 5f) up to 21.65% (Fig. 6). Group (G) demonstrated moderate inflamed granulation tissue occupying the wound gap with epidermal remodeling under necrotic crust at the wound edge (Fig. 4g) as well as an intermediate maturation in the collagen fibers (Fig. 5f) up to 22.64% (Fig. 6). Group (H) demonstrated the same records as group A samples with an intact covering epithelium and an intact dermal layer (Fig. 4h) and significant normally distributed collagen deposition (Fig. 5h) up to 39.3% (Fig. 6).

#### In vitro skin permeation studies (ex vivo analysis)

The permeation analysis indicate that the amounts of drug permeated from ODHP-NS-HG and isoconazole 1% (Candicure®) were 94.47 ± 2 and 60.041 ± 3 µg/cm^2^, respectively, which indicates that the amount permeated from ODHP-NS-HG was significantly different (*p* < 0.05) and 1.6-fold greater than that of isoconazole 1% (Candicure®).

#### Angiogenesis, inflammatory markers, and pro-inflammatory cytokine measurements

**Fig. 7:**
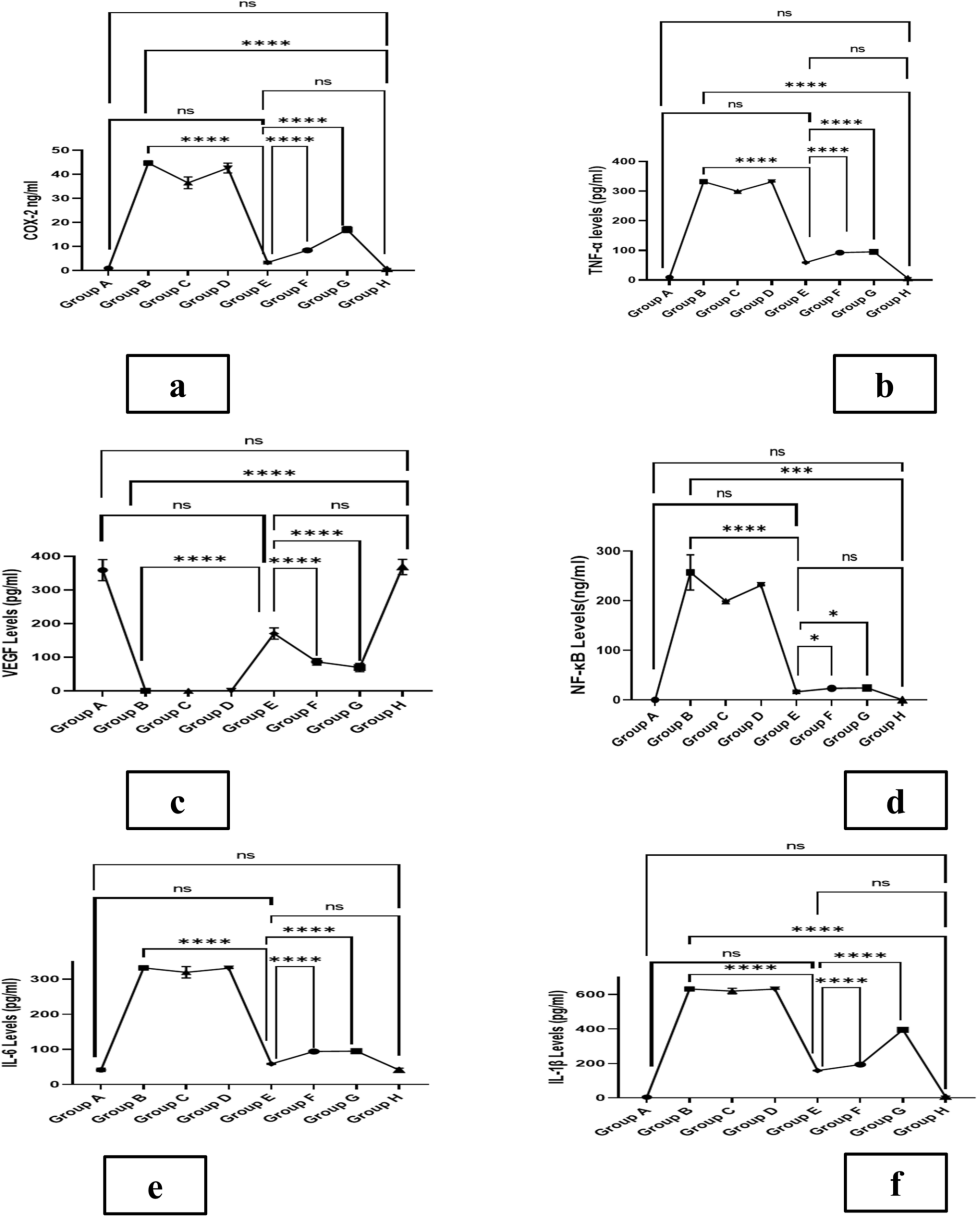
The levels of the respective biomarkers for each group corresponding to the levels of the inflammation recovery. The biomarkers are displayed as follows: (**a**) COX-2, (**b**) TNF-α, (**c**) VEGF, (**d**) NF-kB-p105, (**e**) IL-6, and (**f**) IL-1β

The mean levels of COX-2, TNF-α, NF-κB-p105, IL-6, and IL-1β for group E were 3.95 ± 0.3 ng/mL, 58.608 ± 1 pg/mL, 15.776 ± 3 ng/mL, 58.765 ± 3 pg/mL, and 157.748 ± 15 pg/mL, respectively, which were significantly lower than those of groups C, B, and D (*p* < 0.001), positive control groups (G, F) (*p* < 0.05), and not significantly different than values of groups A and H (*p* > 0.05). The level of vascularization stated by VEGF levels assay was significantly higher in group E (359.178 ± 5 pg/mL) compared to groups C, B, and D (*p* < 0.001), 98-folds higher than group B, 2-folds greater than positive control F (86.528 ± 6 pg/mL), 2.5-folds greater than positive control G (69.356 ± 5) (*p* < 0.05), and not significantly different than those of groups A and H (*p* > 0.05). Figure 7 graphically demonstrates the levels of the respective biomarkers corresponding to the levels of inflammation and angiogenesis as mentioned.

## Discussion

This study was aimed to create a formulation containing the antifungal compound ODHP with the highest quality that would provide the best criteria required for topical application. ODHP is a potent antifungal agent that was previously extracted from *Alcaligenes faecalis* MT332429 culture broth, purified and production optimized using RSM in our previous study (El-Sayed et al. 2020a). Secondary bacterial metabolites are stable and resistant to modification by microorganisms that produce them (El-Sayed et al. 2021). The increasing demand for natural products and bulk chemicals demands sustainable fermentation technologies has identified beneficial soil bacteria as bio-factories that can boost yield while minimizing environmental risks (Bentzon-Tilia et al. 2016). Antimicrobial misuse and resistance are increasing, necessitating the replacement of chemically synthesized antimicrobials with naturally produced ones (Årdal et al. 2020). As a result, industrial microbiology offers environmentally friendly alternatives to fossil fuels, non-biodegradable plastics, and polluting chemical processes, making soil bacteria like *Alcaligenes faecalis* more attractive for sustainable production (Harwood et al. 2018).

Researchers are moving towards nanocarriers with distinctive properties, optimal effectiveness, specificity, and less side effects. One effective solution is nanosponge particles, which are water soluble, can enclose the hydrophobic drug within the nanosponge, provide drug delivery at a targeted site, have less harmful side effects, are stable over a pH range from 1 to 11, are thermostable up to 130 °C, can be used to mask unpleasant flavors, are biodegradable, and transform liquid substances to solids (Potdar et al. 2022). Moreover, particles can be made smaller or bigger by varying the proportion of cross-linker to polymer and have predictable drug release (Pandey 2019). Nanosponges are also self-sterilizable because their typical pore size is 0.25 micron, which bacteria cannot penetrate (Potdar et al. 2022). In order to restrict COVID-19 fungi co-infections, nanosponges have been employed against SARS-CoV-2 and have demonstrated potential inhibitory effects for biological neutralization and antiviral medication delivery applications (Mostafavi et al. 2022). Furthermore, such cellular nanosponges may be useful for biological neutralization of chemical toxic agents, inflammatory cytokines, bacterial toxins, virus fragments, and pathological antibodies (Wang et al. 2022). Other benefits of using NSs include a greater degree of entrapment, which aids NSs to serve as a reservoir for different pharmaceutical substances. They also aid in the protection of compounds from degradation (Tiwari and Bhattacharya 2022). Hence, NSs were used for the delivery of ODHP and were prepared with the emulsion-solvent diffusion method. This method has two advantages: it does not require emulsifiers, and it has a straightforward and simple preparation yielding significant results (ARABI et al. 2023). We used the Box-Behnken design to optimize the process parameters to achieve an effective and economical result (Ahmed et al. 2022). This model of design was successfully employed in previous studies to optimize production of rhamnolipids (El-Housseiny et al. 2016), antibiotics (Ibrahim et al. 2019; El-Housseiny et al. 2021), and antifungal metabolites by their producing isolates (El-Sayed et al. 2023, 2020b). Measurement of entrapment efficiency can be performed indirectly where the amount of free unentrapped drug is measured spectrophotometrically at the corresponding wavelength (i.e., *λ*_max_), and thus the entrapped amount of drug can be calculated according to Fahmy et al. (2023) by subtracting the drug content in the supernatant (after the first and second centrifugations) from the initial amount of drug added to the nano-system and dividing it by the total drug content. Alternatively, entrapment efficiency can be directly calculated via disrupting the membranes of the developed nanoparticles to allow for the release of the entrapped drug (for spectrophotometric detection) utilizing organic solvents (e.g., methanol) or Triton™ X-100 and measured as mentioned previously (Penjuri et al. 2016). However, the direct method does not take into consideration the amount of non-entrapped drug to allow more clear comparison, while the indirect methods could only be used during preparation process and could not be applied after lyophilization (Amini et al. 2017). The entrapment efficiency was previously reported to increase along with the homogenization period and speed to a considerable extent (Rossi et al. 2014). In correlation with previous reports, the decreased cross-linker concentration resulted in reduced entrapment efficiency, suggesting that at low concentrations, there may have been fewer sites for drug complexation available due to insufficient meshwork inside the polymer (Srivastava et al. 2021). However diphenyl carbonate may have destroyed the intermolecular hydrogen bond network of discrete units of β-CD at greater concentrations as previously reported by Mavila et al. (2016). Hence, it was deemed necessary to optimize the process parameters to compromise and get the suitable ratio between β-CD and diphenyl carbonate. Particle size (PS), polydispersity index (PDI), and entrapment efficiency % (EE%) were considered the dependent process variables and influenced the formation of ODHP-NS in agreement with previous studies (Ghose et al. 2020; Rehman et al. 2021; Srivastava et al. 2021). Upon characterization, the optimized ODHP-NS has a particle size of 332 ± 0.35 nm. It was found to be homogenous and had a porous surface and spherical morphology with the drug entrapped in the polymer matrix according to the SEM image. SEM is a robust technique for displaying the sample’s morphology, providing information on the sample’s pore structure and an indication of homogeneity (Datye and DeLaRiva 2023).

The primary indicator for the stability of the colloidal dispersion is zeta potential. The zeta potential can be determined by adding an additional electrode to particle size analysis equipment or a zeta seizer. ODHP-NS has a mean ZP of − 13.8 ± 0.56 mV, indicating a relatively good stability and dispersion quality (Ahmed et al. 2021; Penjuri et al. 2016). This is in agreement with studies which reported nanosponges with comparable negative zeta potential values and which produced stable water suspensions that do not undergo aggregation over time (Kumar et al. 2021; Trotta et al. 2012). The more stable a colloidal dispersion is, the higher its zeta potential value, positive or negative (Bhowmik et al. 2018). The “polydispersity index” (PDI) is a measure used to specify the size range of the NS systems regarding particle size distribution characterization. The degree of non-uniformity in a particle size distribution is referred to as “polydispersity” (Bera 2015; Kumar et al. 2021). Because of the dimensionlessness and scale of this measure, values lower than 0.05 are often observed only with extremely monodisperse standards (Bera 2015). ODHP-NS has a PDI of 0.233 ± 0.02 showing reasonable and acceptable monodisperse system. A sample with a very wide particle size distribution and a PDI value greater than 0.7 is most likely not appropriate for analysis using the dynamic light scattering (DLS) method (Danaei et al. 2018). The entrapment efficiency was found to be in an acceptable range of 90.14 ± 0.65% which was further corroborated from earlier study (Amer et al. 2020). The drug loading was found to be 87.5%. The manner of drug loading into the nanosponges can influence the drug-nanosponge complexation. Although the efficacy of an approach is mostly determined by the nature or features of the drug and polymer, freeze-drying has been reported to alter drug and nanosponge complexation in some situations (Bhowmik et al. 2018). ODHP-NS formulation has attained high drug loading capacity; this is in accordance with many studies stated that cyclodextrin-based nanoparticles can maintain a high loading capacity of various small molecules for drug delivery (Deng et al. 2021). Nanosponges have a higher drug loading capacity than other nanocarriers, making them ideal for overcoming issues such as active drug stability, solubility, and delayed release (Tejashri et al. 2013). Differential scanning calorimetry (DSC) measures the heat which is being absorbed or released during the process of heating or cooling. It is used to measure the heat of reaction, melting point, heat capacity, and glass transition (Akash et al. 2020). DSC of optimized ODHP-loaded NS ensured its development which leads to the reduction in crystallinity of the drug. The change in the structure from crystalline to amorphous established the fact that NS has been developed (Abdellatif et al. 2017). The molecularly dispersed phase of ODHP within the NS structure leads to the broadening of the peak. The amorphous structure of the ODHP-NS established by the result obtained is desirable for enhanced drug entrapment within the NS structure (Fatima et al. 2019).

Several gel formers have been preliminarily tested for the preparation of hydrogel of which carbopol 940 was selected. Carbopol 940 is a hydrophilic polyacrylic acid polymer with carboxyl functional groups that are ionized after interaction with triethanolamine. This results in the formation of a gel-like structure due to electrostatic repulsion between charged polymer strands. This stage increases the pH of the HG developed, making it suitable for skin application. Additionally, because carbopol is non-toxic and non-irritating, it can be used in gel preparations (Safitri et al. 2020). Unlike hydroxypropyl methylcellulose, which must be produced in hot water, carbopol gelling agents have the advantage of being able to be developed in room temperature water. Furthermore, the wide viscosity range of 40,000–60,000 cps of carbopol 940 led to its selection for hydrogel preparations (Daryab et al. 2022). The concentration of carbopol 940 gelling agent directly influences the viscosity of the preparation, which also influences its physical characteristics (Daood et al. 2019). Good spreadability is crucial for acceptable gel formulation. HG’s spreadability was found to be 5.74 ± 0. 32 cm, and pH was 6.75 ± 0.13. The HG’s pH, which was nearly neutral, indicated an absence of skin irritation potential, thus verifying its compatibility with the skin (Blaak and Staib 2018). The swelling data can be employed to determine the mechanical and viscoelastic properties, degradation rate, cross-linking degree, and refractive index (Sievers et al. 2021). The gel exhibited a favorable swelling index of 350.6 ± 0.66%, indicating good swelling characteristics that align with previous findings (Ambala and Vemula 2015). While the extrudability was recorded as 0.97 ± 0.11 (g/s), the viscosity of hydrogel was found to be 1092 ± 4cps which indicates good consistency and texture of the prepared hydrogel. The viscosity of hydrogel compositions reflects their consistency in general (Dejeu et al. 2022).

The percentage of drug content for the selected HG-5 was 90.96 ± 0.32% denoting high retention of the drug by the nanosponge system. Based on the coefficient of correlation (*R*^2^ value = 0.9976), the release was best fitted into the Higuchi diffusion kinetic model. This model is based on several hypotheses, including that initial drug concentration in the matrix is substantially higher than drug solubility, matrix swelling and dissolution are minimal or non-existent, and drug diffusivity is constant (Wang et al. 2023). The Korsmeyer-Peppas model was used to investigate the mechanism of ODHP release from the produced formula. A diffusion exponent (*n* = 0.563) reflected a non-Fickian model (anomalous transport) in which release is governed by a combination of diffusion and polymer chain relaxation (Yammine et al. 2023). The drug content was reassessed following exposure to UV light, as well as after conducting stability studies under the specified conditions. The results indicated that there was no significant difference (*p* > 0.05) compared to the initial conditions, indicating the formulation’s exceptional stability properties. In vitro antifungal study demonstrated superior antifungal activity of ODHP-NS-HG against *C. albicans* ATCC10231 over the positive control fluconazole.

Since its introduction by Mosmann in the 1980s, the MTT assay has become the gold standard for determining cell cytotoxicity (Hoogstraten et al. 2021). IC_50_ values of the optimized ODHP-NS and fluconazole (as reference standard) were 325.6 µg/mL and 287.8 µg/mL, respectively. Cell viability was reduced by both of these samples in a dose-dependent manner (Van Tonder et al. 2015). Finally, a thermal injury model in male Wistar albino rats infected with *C. albicans* was developed to assess the capability of the ODHP-NS designed as hydrogels to suppress the pathogenicity of this strain in rats. Many studies using *C. albicans* superficial skin infection in mice/rats have been described earlier (El-Sakhawy et al. 2023; Macherla et al. 2012). Our results revealed that the tested formulation of ODHP-NS-HG increased the survival rate, as well as increasing the deposition of dermal collagen fibers in the skin of treated groups compared to the other control groups. This is in agreement with the previous study investigating the level of collagen deposition and the healing of *C. albicans*–infected wound (Nejati et al. 2015). According to another study, it was proposed that the effectiveness of antifungal agents in the form of nanosponges could be enhanced by β-CD. This is because when β-CD interacts with *C. albicans*, it induces modifications in the cell wall and disrupts its protective function (Finger et al. 2013). Additionally, since *C. albicans* contains glucosylceramides in its cell wall, which activate an internal signaling pathway leading to the apoptosis of the fungal pathogen, it may have a greater tolerance to higher concentrations of β-CD compared to other microbes. This allows first-line therapies for virulent and highly resistant fungal infections to use antifungal agents formulated as β-CD-NS without the side effects of synthetic and semi-synthetic drugs (Desai and Shende 2021). The wound healing process necessitates integrated and sequential stages to repair cells and tissues and return them to their pre-injury state (Behere and Ingavle 2022), and fungal contamination can delay the process of wound healing. The wound area and percentage of wound contraction were observed to assess the ODHP-NS-HG ability to cure wounds. In the current research, wound areas and wound contraction (%) were measured at days 1, 7, and 14 after the wound was created.

According to the study’s results, ODHP-NS-HG can help treat wounds infected with *C. albicans* and speed up the healing process. It is worth noting that the proportion of wound contraction in ODHP-NS-HG–treated group was found to be 80% which was 1.5-fold higher compared to positive control group (F) and 2.4-fold greater than positive control group (G). Our study appears to be the first to investigate the efficacy of ODHP-NS-HG for the treatment of candidiasis in a rat thermal injury model. The histopathological inspection of various injured groups revealed varying degrees of tissue damage and healing mechanisms. Isoconazole 1% along with collagenase were used as positive controls in our research for the treatment of rat skin wounds, as described in a couple of studies (Durmus et al. 2009; Veraldi 2013) owing to their wound healing properties. In comparison to the control group B (burned, infected, untreated), topical application of ODHP-NS-HG in group (E) showed a significant difference (*p* < 0.05) in minimizing local wound infection and promoting rejuvenation of the skin, as well as higher records and a remarkable acceleration of dermal mature collagen fiber development. Thus, our findings support a previous report by Srivastava et al. that nanosponges of antifungal therapies could be promising in preventing wound-associated *C. albicans* infections (Srivastava et al. 2021). The efficient dermal delivery of ODHP-NS through skin in topical gel formulation was confirmed via high drug amount in the in vitro permeation study.

Dermatomycoses, or superficial fungal skin diseases, are caused by a variety of pathogens, including dermatophytes, yeasts, and molds (Costa-Orlandi et al. 2023; Jaishi et al. 2022). Because of exo-enzymes secreted by the fungal pathogen at the site of infection, such as keratinase (Veraldi 2013), inflammatory symptoms and indications, such as pruritus and erythema, are very common (AbouSamra et al. 2020). Hence, it was deemed necessary to estimate the levels of angiogenesis (VEGF), inflammatory markers like cyclooxygenase (COX-2) and pro-inflammatory cytokines (TNF-α), NF-κB-p105, IL-6, and IL-1β based on ELISA technique. Angiogenesis, or the creation of new blood vessels, is critical in wound healing (Ahmad and Nawaz 2022). The most significant and well-investigated angiogenic factor is vascular endothelial growth factor (VEGF) (Honnegowda et al. 2015). Many researchers believe that VEGF promotes wound epithelialization and collagen deposition so that high levels of VEGF are produced during the normal wound repair (Chereddy et al. 2015). ODHP-NS-HG–treated group (E) exhibited significantly (*p* < 0.05) higher level of VEGF compared to the infected untreated group (B) denoting increased level of re-epithelization and collagen deposition in accordance with previous studies (Abdel-Motaal et al. 2022). Cyclooxygenases are essential enzymes in the production of prostaglandins (PGs) from arachidonic acid. While COX-1 is constitutively expressed, COX-2 is activated in injured tissues, resulting in inflammatory processes (Carvalho et al. 2022). COX-2 inhibition may result in anti-inflammatory effects (Ju et al. 2022). Our ODHP-NS-HG formulation has significantly decreased the levels of COX-2 compared to the infected untreated group (B) implying its additional anti-inflammatory effect along with its antifungal one (Das et al. 2011; López et al. 2011). In the injured region, tumor necrosis factor (TNF)-α is rapidly produced, causing inflammation in wound tissues (Jaishi et al. 2022). Hence, TNF-α was elevated in infected untreated groups while it was neutralized significantly in ODHP-NS-HG–treated group E indicating good healing of the injured area. During wound healing, the classical NF-κB pathway is activated, resulting in the production of numerous cytokines, and other secondary inflammatory mediators, and apoptosis inhibitors (Hellweg 2015).

On the other hand, over expression of NF-κB can lead to reduced wound healing as previously confirmed (Jin et al. 2021). Based on these facts, our ODHP-NS-HG formulation showed decreased levels (*p* < 0.05) of NF-κB denoting healing of the wound compared to other untreated groups that showed higher levels denoting that the wound was still in the healing process. IL-6 has potential roles in the wound healing process as previously reported (Johnson et al. 2020). Decreased levels of IL-6 in group E compared to untreated groups (*p* < 0.05) indicate a normal wound repair. This comes in accordance with the observations from other reports (Johnson et al. 2020). IL-1β induces fibroblast growth, promotes the production of collagenase, and inhibits endothelial cell development, making it an antagonist of extracellular matrix metabolism (Carvalho et al. 2022). Furthermore, IL-1β has been shown to promote the proliferation of smooth muscle cells and to serve as a chemoattractant for neutrophils and macrophages (Paramel et al. 2022). According to our study’s ELISA findings, IL-1β levels were higher in the untreated groups than in the treated ones. This indicates that ODHP-NS-HG application is effective at inhibiting inflammation via IL-1β, which is consistent with the findings of Aneesha et al. (2022) and Gürgen et al. (2014). The substantial reductions in COX-2 and pro-inflammatory cytokines found in group E indicate that ODHP-NS-HG speeds up the healing process by inhibiting the inflammation phase. In conclusion, this research demonstrates the effective optimized preparation of nanosponges from the antifungal ODHP. The developed NS was characterized for particle size, PDI, zeta potential, and FTIR, and based on the findings, a topical hydrogel formulation was prepared. Upon evaluation, the hydrogel had excellent spreadability, extrudability, and swelling properties. The hydrogel pH confirmed its skin compatibility, and it showed high drug content with potent in vitro antimycotic activity. The in vitro release studies were best fitted to Higuchi’s model. Furthermore, the stability studies denoted the high stability of this formulation. Further in vitro, ex vivo, and in vivo testing revealed that the prepared hydrogel inhibited fungal growth both and showed enhanced permeability following its topical application. The prepared ODHP-NS-HG reverted the inflammatory effect and suppressed the infection by compromising the levels of COX-2, TNF-α, NF-κB-p105, IL-6, and IL-1β in treated group E comparing to untreated groups. Moreover, VEGF levels were significantly higher in treated group E indicating higher level of vascularization than untreated groups.

## Supplementary Information

Below is the link to the electronic supplementary material.

Supplementary file1 (PDF 1107 KB)

## Data Availability

The authors declare that the data supporting the findings of this study are available within the article and its supplementary information file. The 16S ribosomal RNA of *Alcaligenes faecalis* MT332429 deposited into the NCBI GenBank under the accession number MT332429 (https://www.ncbi.nlm.nih.gov/nuccore/MT332429) (accessed on 23 July 2023).
