# Supplementary material for: Nanosponge hydrogel of octadecyl 3-(3,5-di-tert-butyl-4-hydroxyphenyl) propanoate of Alcaligenes faecalis
Source: Appl Microbiol Biotechnol. 2024 Jan 12;108:100. doi: 10.1007/s00253-023-12819-3 (PMC10786974; doi:10.1007/s00253-023-12819-3)
Supplement: Supplementary file 1 [file 253_2023_12819_MOESM1_ESM.pdf]

Nanosponge-Hydrogel of Octadecyl 3-(3, 5-di-tert-butyl-4-hydroxyphenyl) propanoate of *Alcaligenes faecalis*

Sayed E. El-Sayed[1], Neveen A. Abdelaziz[1,] Ghadir S. El-Housseiny[2], Khaled M. Aboshanab[2]*

[1]Department of Microbiology and Immunology, Faculty of Pharmacy, Ahram Canadian University, Sixth of October City, Giza 12451, Egypt

[2]Department of Microbiology and Immunology, Faculty of Pharmacy, Ain Shams University, Organization of African Unity St., Abbassia, Cairo 11566, Egypt

* **Corresponding Author: Khaled M. Aboshanab**

Address: Department of Microbiology and Immunology, Faculty of Pharmacy, Ain Shams University, Organization of African Unity St., Abbassia, Cairo 11566, Egypt.

E-mail: aboshanab2012@pharma.asu.edu.eg

Mobile: +201-0075-82620

Fax: (202)24051107

https://orchid.org/0000-0002-7608-850X

Authors' e-mails

Sayed E. El-Sayed: sayed.emad@acu.edu.eg

Khaled M. Aboshanab: aboshanab2012@pharma.asu.edu.eg

Neveen A. Abdelaziz: neveen.abdelaziz@acu.edu.eg

Ghadir S. El-Housseiny: ghadir.elhossaieny@pharma.asu.edu.eg

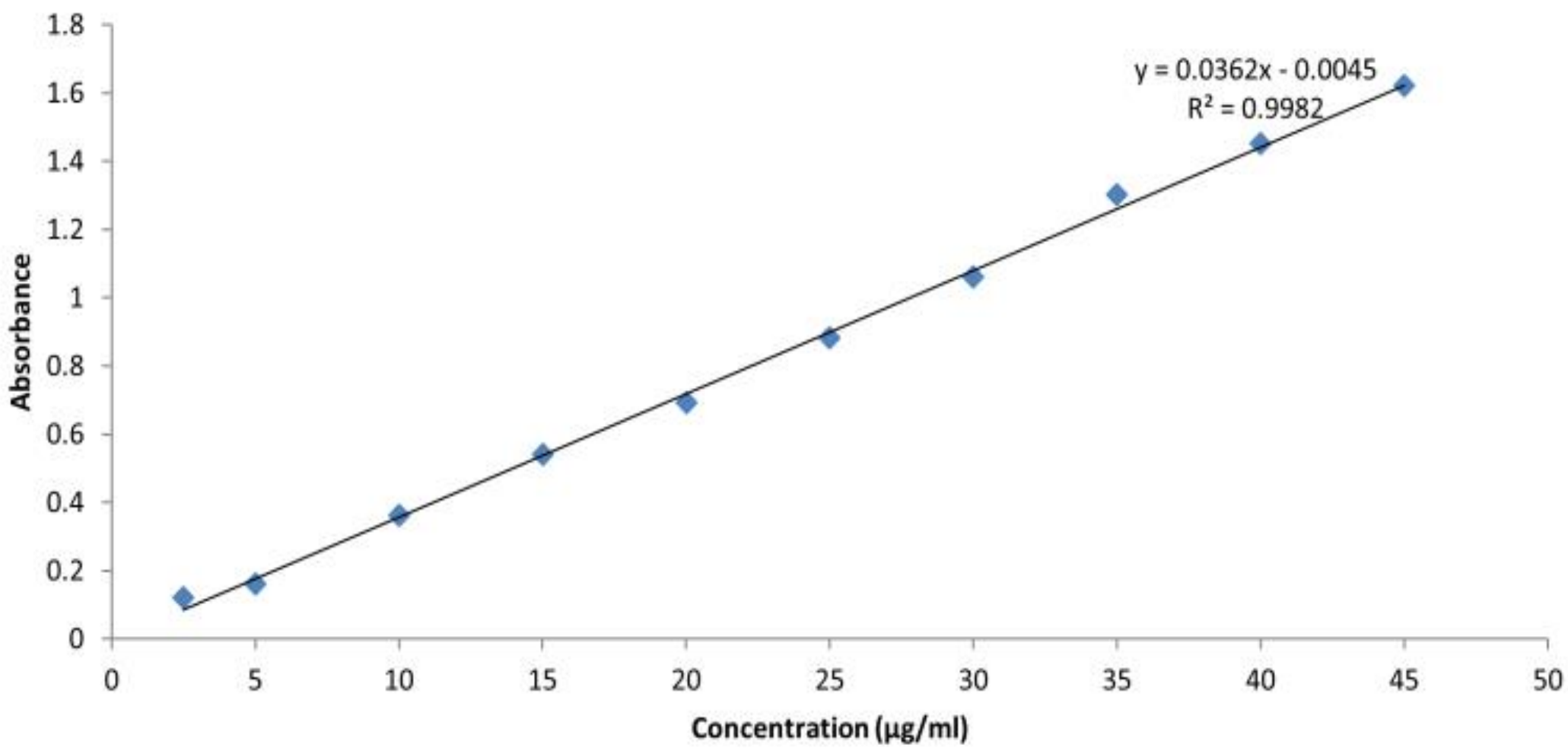

Fig.S1 Standard curve of ODHP

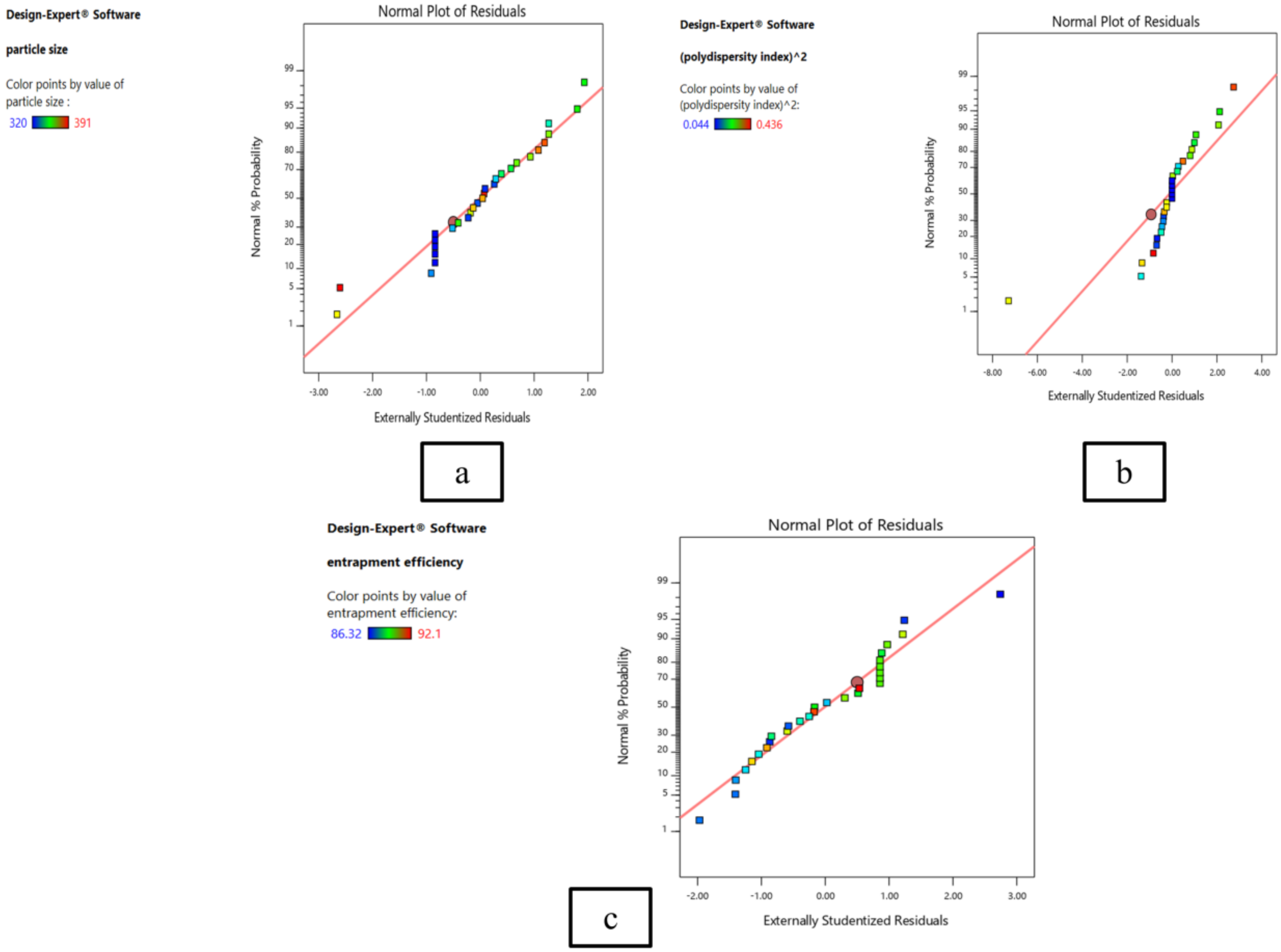

Fig.S2 The normal probability plots of residuals for (a) PS, (b) PDI, and (c) EE%

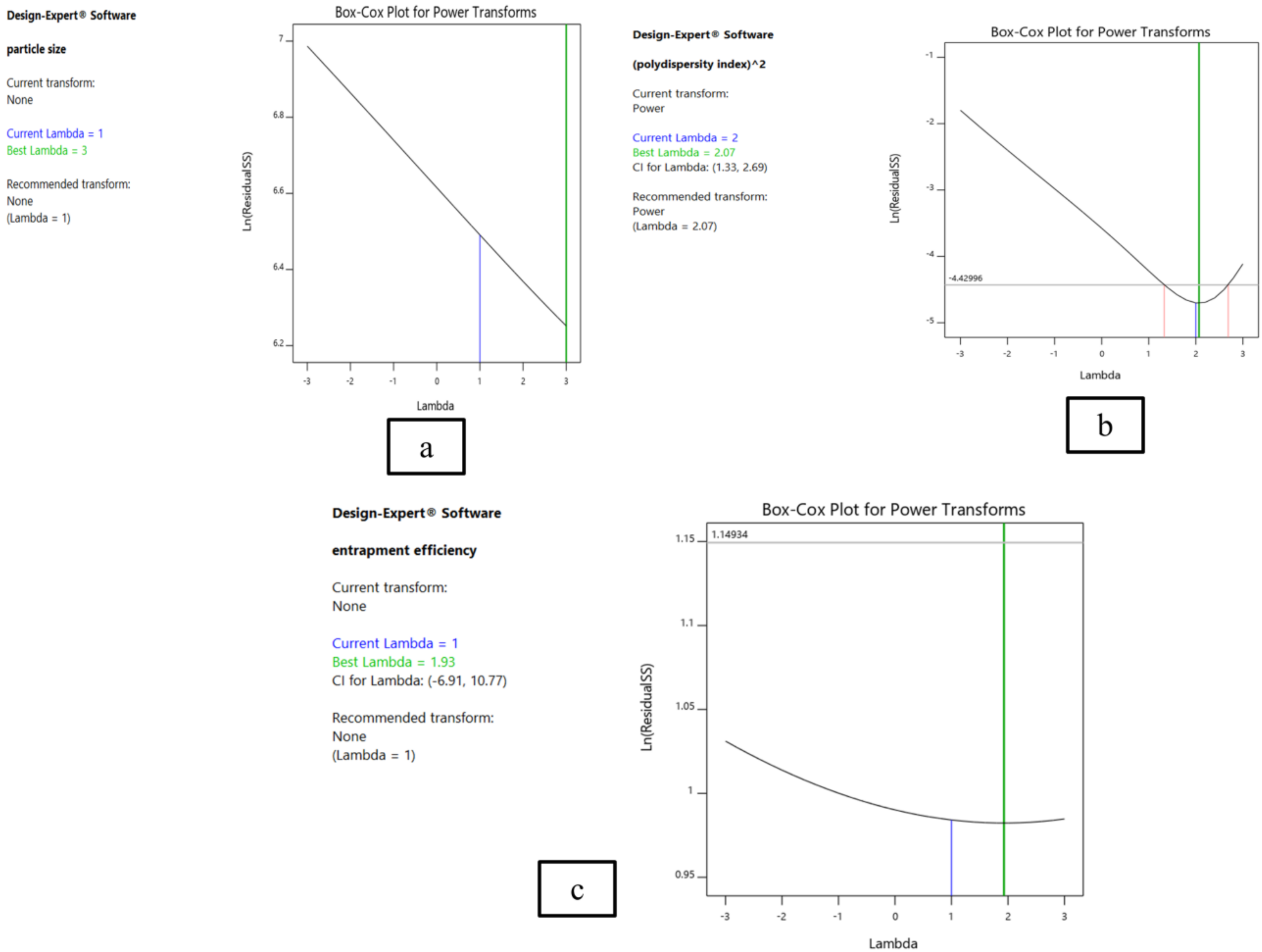

Fig.S3 The Box Cox plots of (a) PS, (b) PDI, and (c) EE%

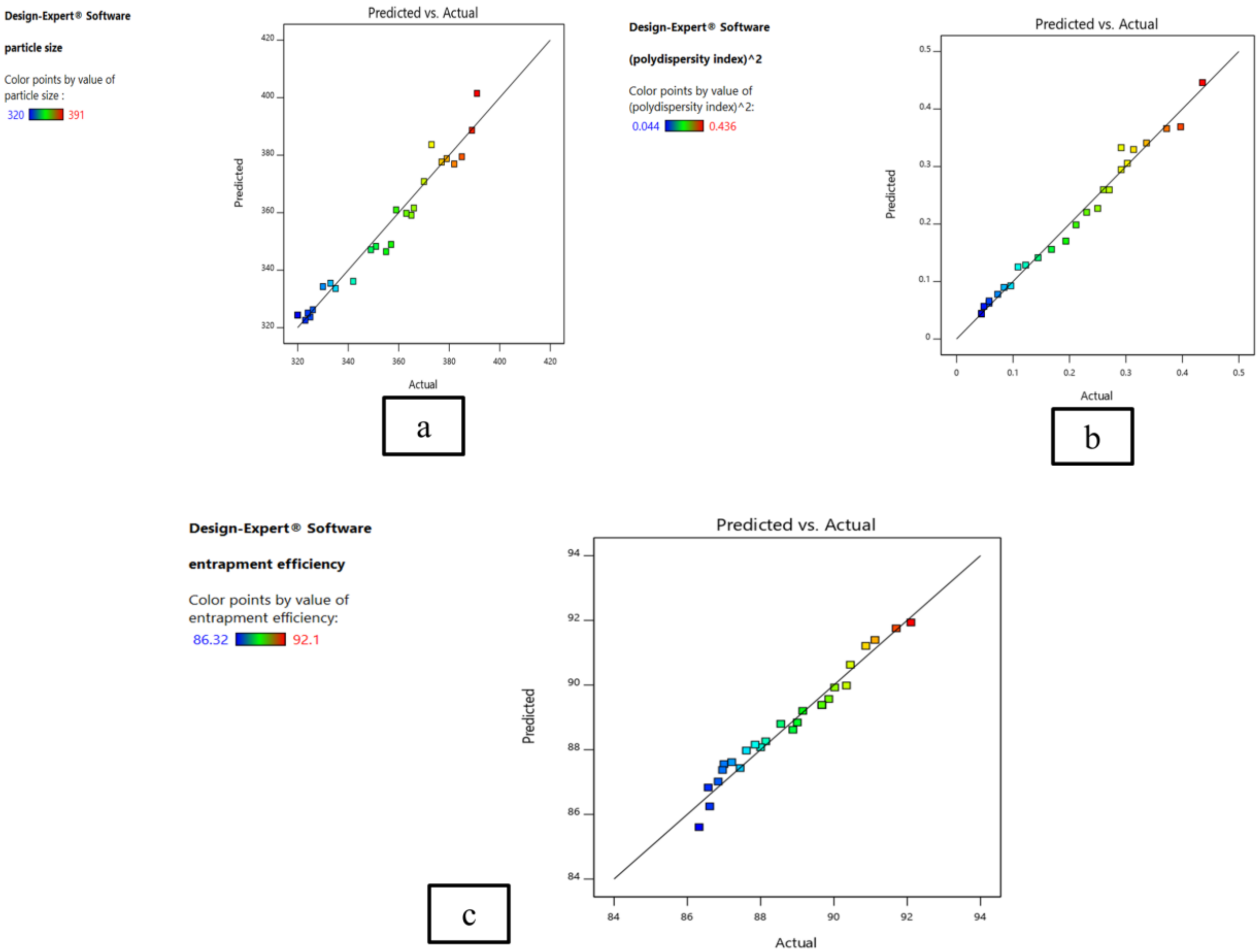

Fig.S4 The predicted versus actual values plot of (a) PS, (b) PDI, and (c) EE%

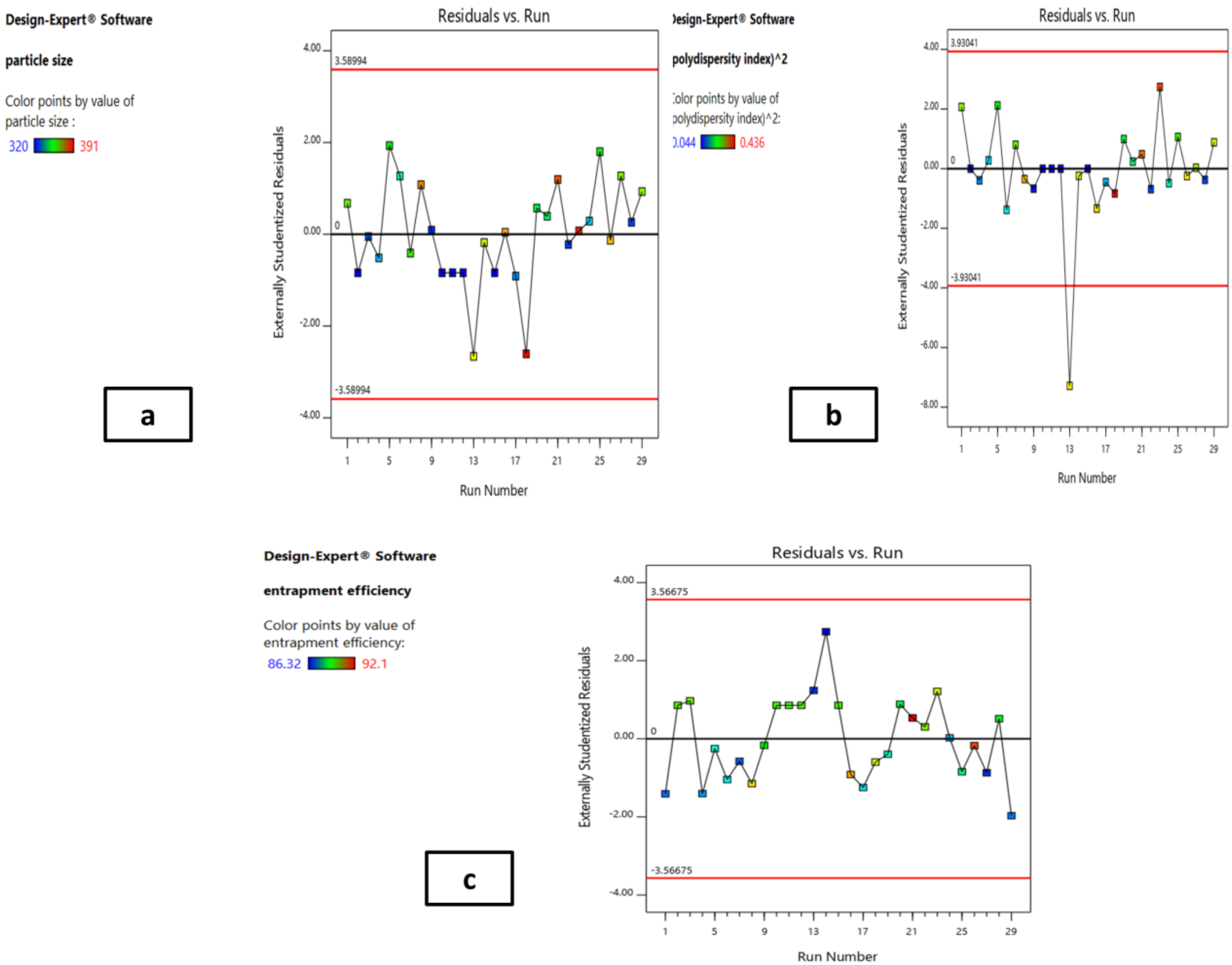

Fig.S5 The residuals versus Run number plot of (a) PS, (b) PDI, and (c) EE%

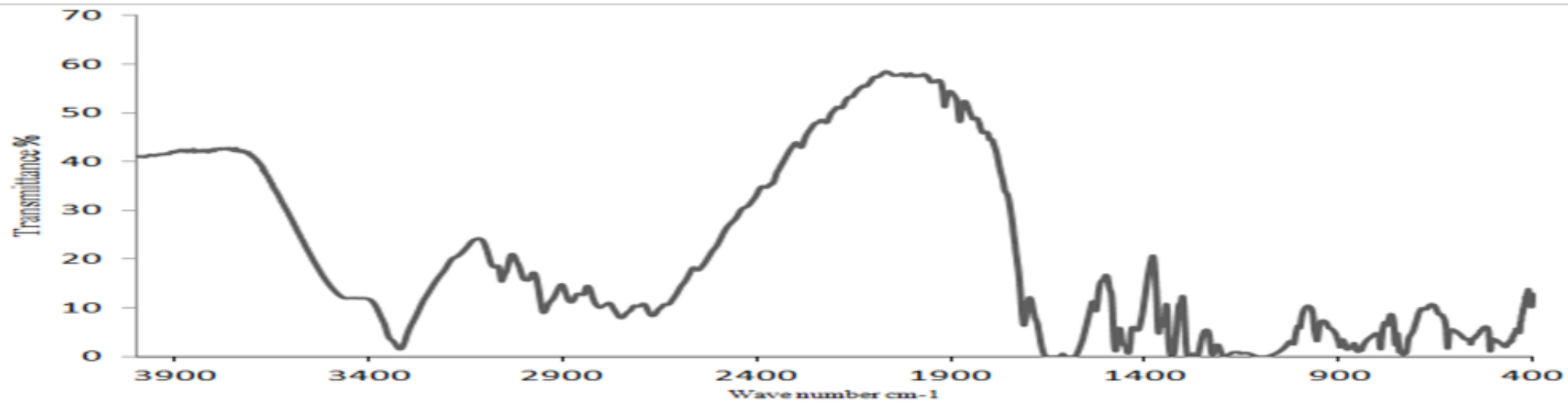

Transmittance %
Wave number cm-1
70
60
50
40
30
20
10
0
3900
3400
2900
2400
1900
1400
900
400

(a)

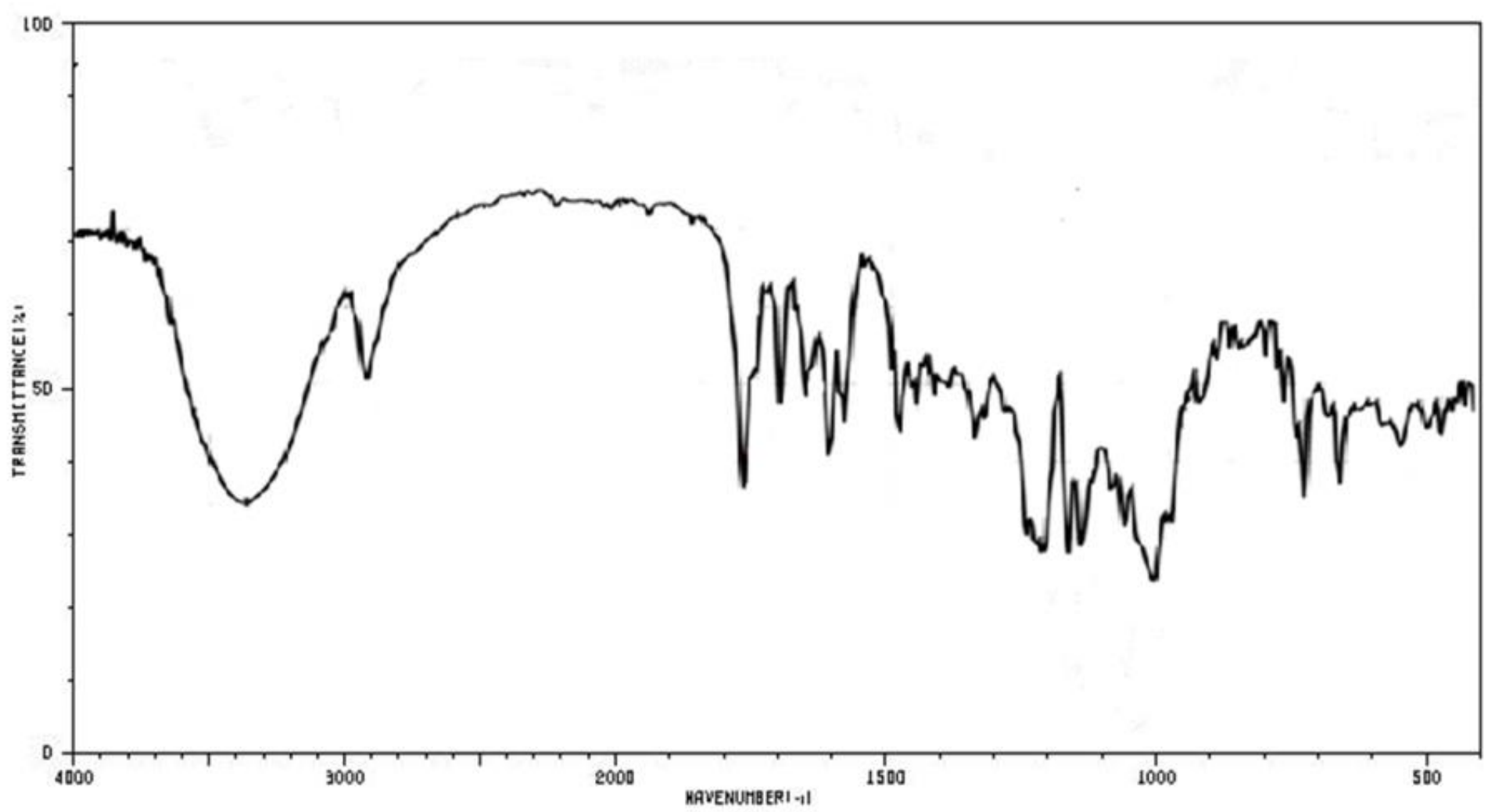

TRANSMITTANCE(%)
WAVENUMBER(-1)
100
50
0
4000
3000
2000
1500
1000
500

(b)

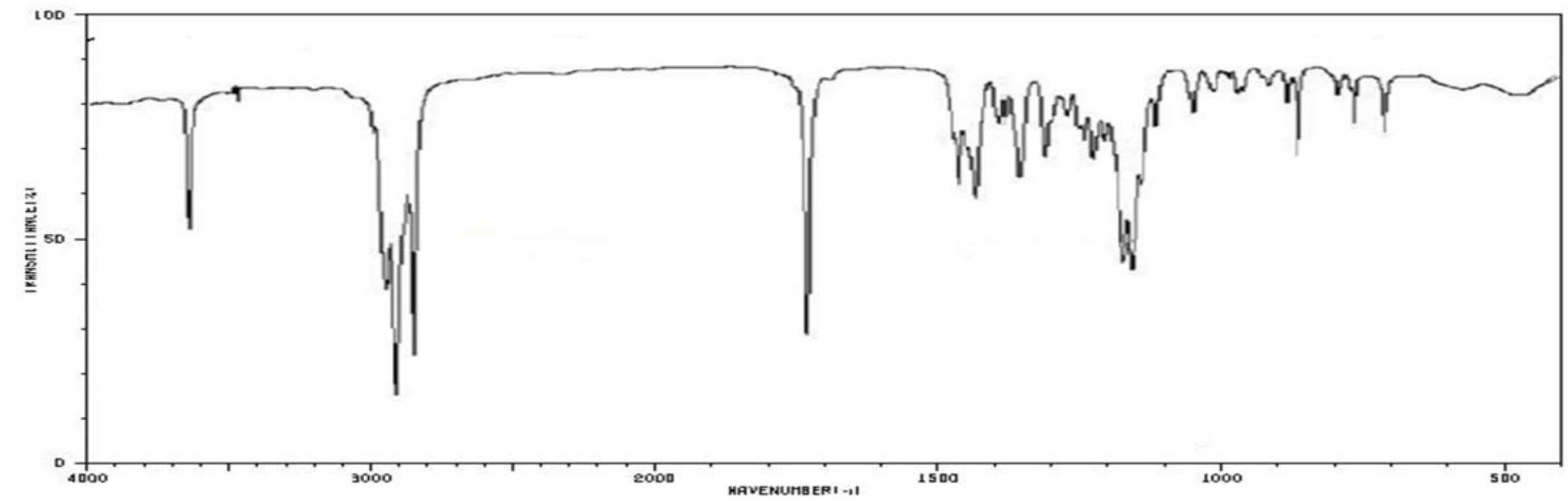

(c)

Fig.S6 FTIR spectra of (a) ODHP-NS complex (b) β-CD and (c) ODHP

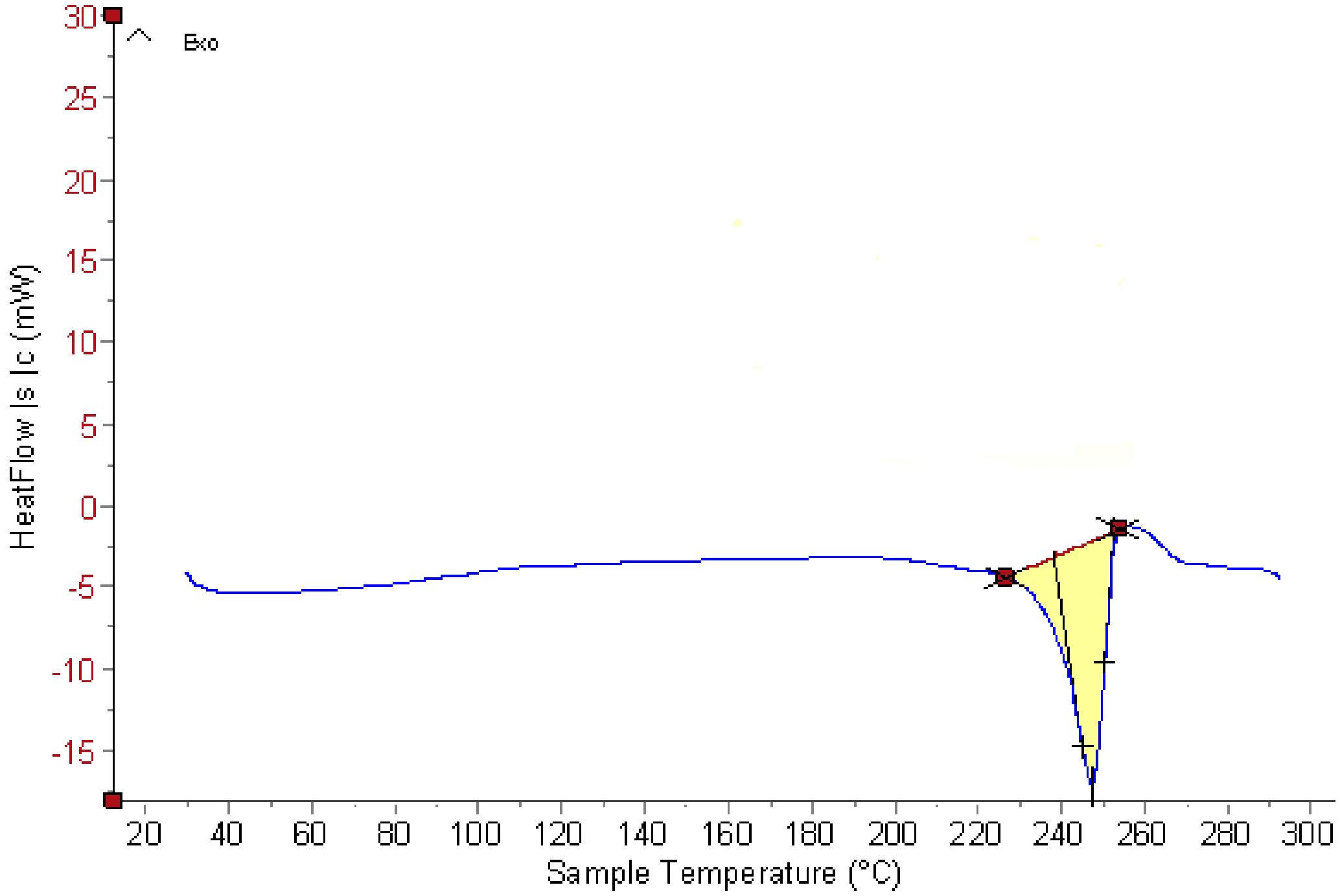

(a)

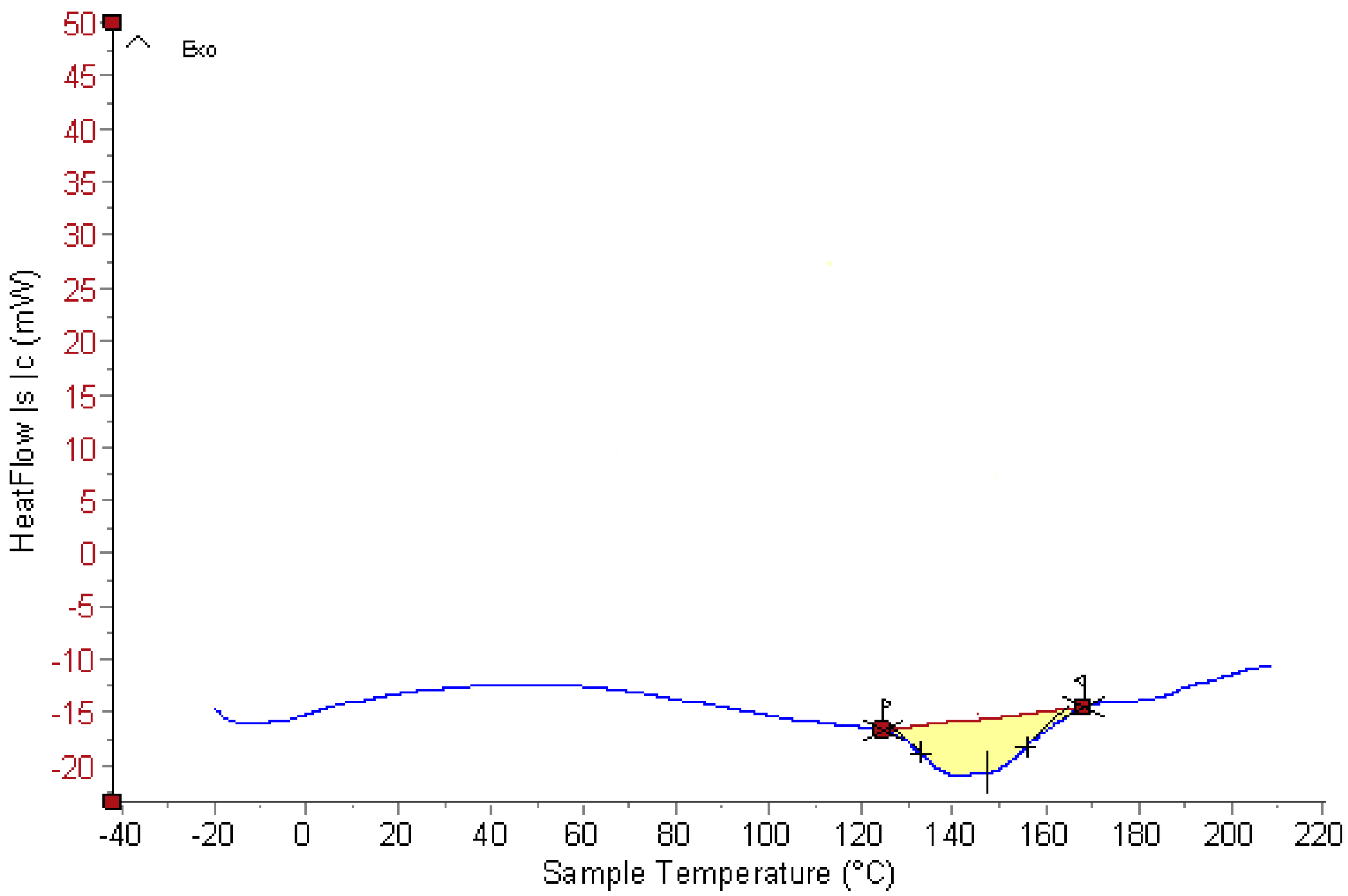

(b)

Fig.S7 Differential scanning calorimetry thermograms of (a) pure ODHP (b) optimized ODHP-loaded nanosponge formulation

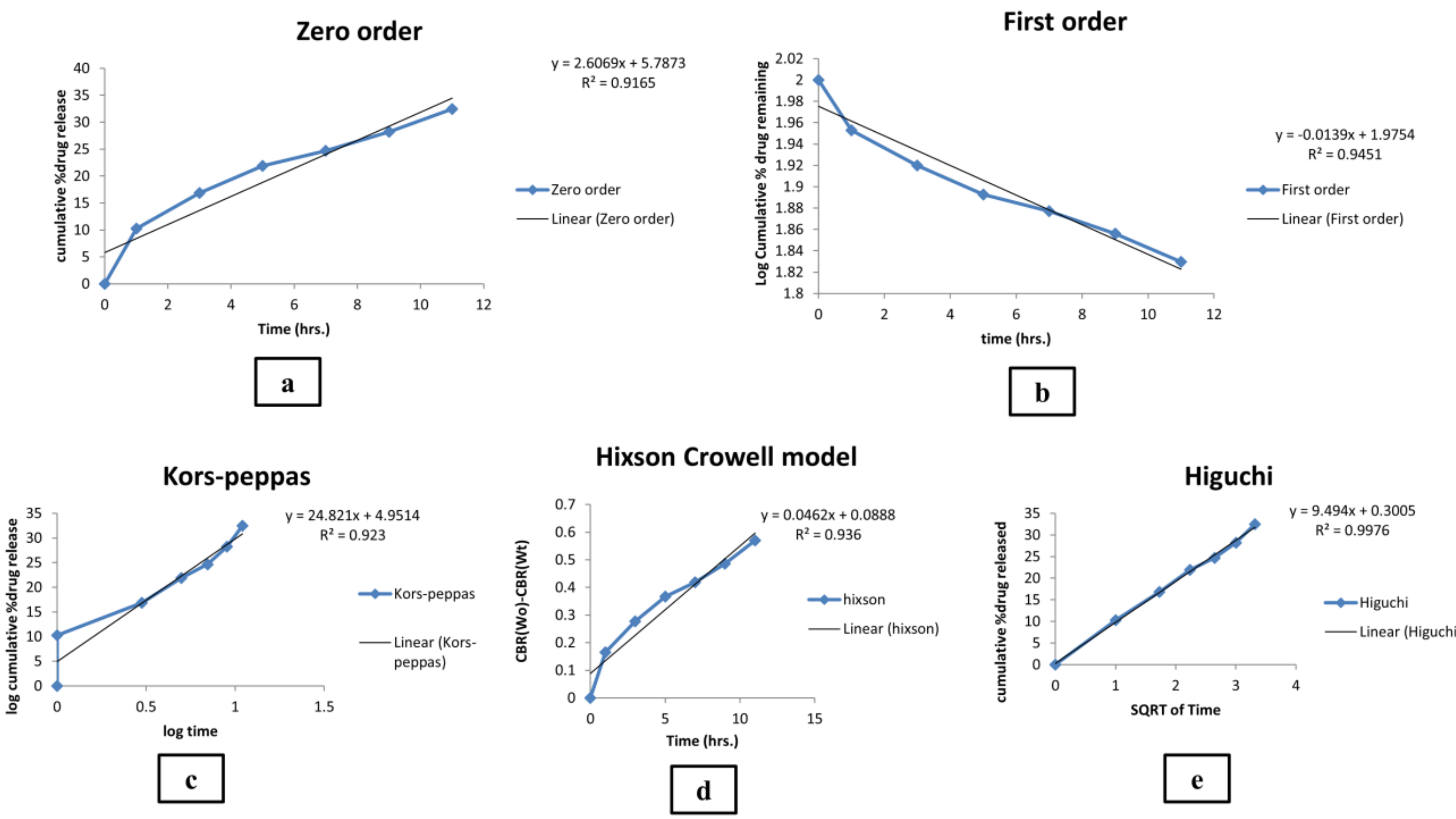

Fig.S8 Various kinetic models of the in-vitro release data **(a)** Zero order, **(b)** First order, **(c)** Korsmeyer and Peppas, **(d)** Hixson and Crowell model and **(e)** Higuchi ' s model

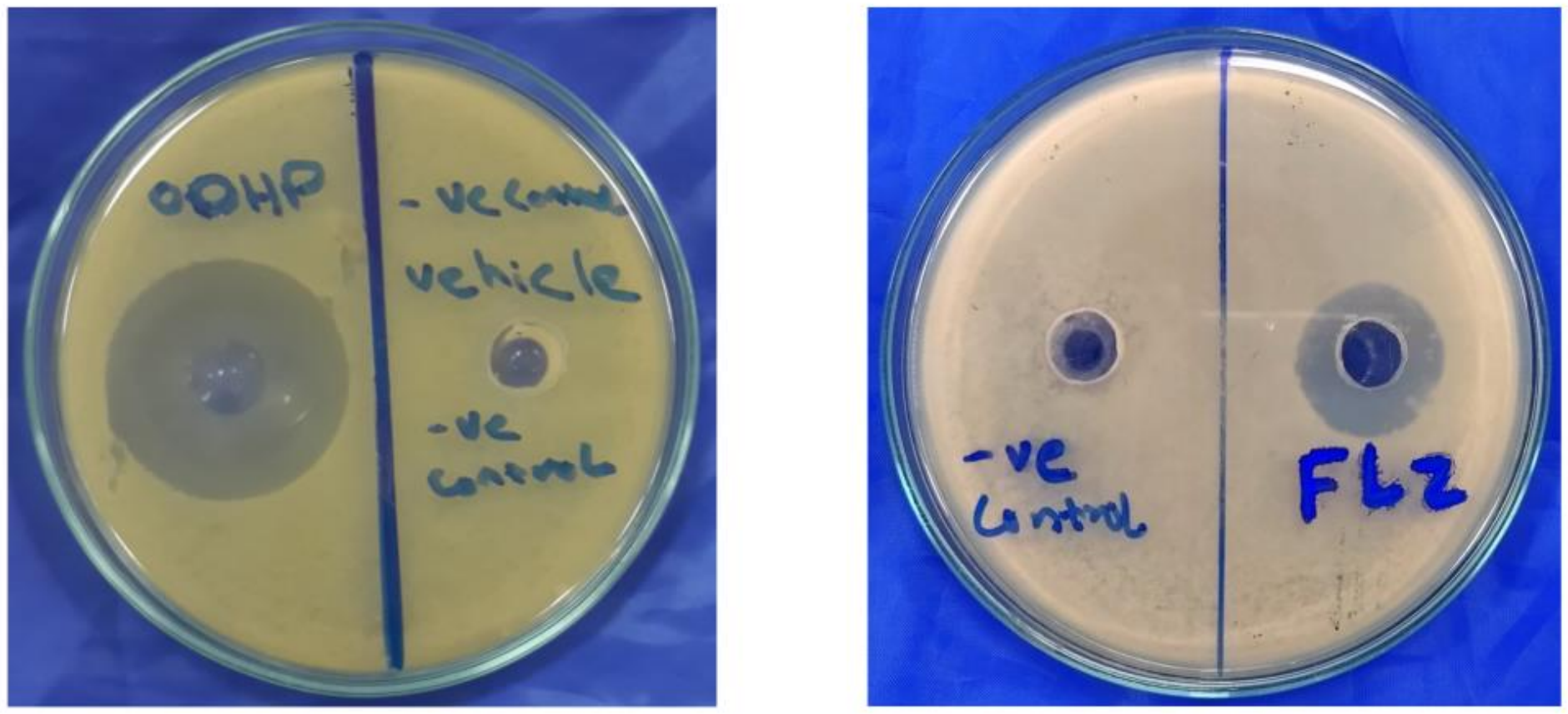

a b

Fig.S9 In vitro antifungal activity of (a) ODHP-NS-HG (b) Fluconazole (FLZ) against *Candida albicans* ATCC10231

Table S1 Evaluation of different gel formers for the preparation of hydrogel

Visual characteristics	Sodium alginate	CMC	Poloxamer 188	HPMC E4	Carbopol 940
Grittiness	Yes (++++)	NO	Yes (+++)	Yes (++)	NO
Stickiness	Yes (++++)	NO	Yes (++)	Yes (++)	NO
Spreadability	Very low (++++)	VERY GOOD	Fairly good	Low (++)	EXCELLENT
Formation of clumps	Yes (+++++)	Low	NO	NO	NO

Table S2 Different gel compositions of carbopol 940

Component	Formulation code					
	HG-1	HG-2	HG-3	HG-4	HG-5	HG-6
Carbopol 940 (w/v %)	0.2	0.5	0.8	1	1.2	1.5
Propylene glycol: distilled water ratio (ml)	30:70	30:70	30:70	30:70	30:70	30:70
Triethanolamine (ml)	2	2	2	2	2	2
ODHP -NS	0.15mg	0.15mg	0.15mg	0.15mg	0.15mg	0.15mg
Methyl paraben (gram)	1	1	1	1	1	1
Distilled water (w/w %) (q.s)	100	100	100	100	100	100

Table S3 Evaluation of different carbopol 940 hydrogel formulations

Visual characteristics	HG1	HG2	HG3	HG4	HG5	HG6
**Grittiness**	Yes (+++)	NO	Yes (+)	NO	NO	NO
**Stickiness**	Yes (+++)	NO	Yes (++)	NO	NO	NO
**Spreadability**	Bad	Bad	Fairly good	Good	Good	Good
**Homogeneity**	CLUMPS	YES	YES	NO	Yes	NO
**Syneresis**	NO	NO	NO	NO	NO	NO
**Color**	White	Translucent	Translucent	Translucent	Translucent	Translucent

Table S4 Comparison of correlation coefficient (R^2) of different kinetic models

	**Zero order**	**First order**	**Korsmeyer and Peppas**	**Hixson and Crowell model**	**Higuchi ' s model**
$\mathbf{R^2}$	0.9165	0.9451	0.923	0.936	0.9976

Table S5 Stability studies data

Storage conditions	Physical appearance	Mean data ± (SD)		
		**pH**	**Viscosity (cps)**	**Drug content**
**Initial**	Translucent-smooth homogenous	6.75±0.13	1092±4	90.96±0.32%
**Room temperature**	Translucent -smooth homogenous	6.92±0.1	1060±5	86.07±0.16%
**5 ± 3 °C**	Translucent -smooth homogenous	6.80±0.02	1185±10	89.41±0.58%
