# Supplementary material for: Nanosponge hydrogel of octadecyl 3-(3,5-di-tert-butyl-4-hydroxyphenyl) propanoate of Alcaligenes faecalis
Source: Appl Microbiol Biotechnol. 2024 Jan 12;108(1):100. doi: 10.1007/s00253-023-12819-3 (PMC10786974; doi:10.1007/s00253-023-12819-3)
Supplement: Supplementary file 1 — Supplementary file1 (PDF 1107 KB) [file 253_2023_12819_MOESM1_ESM.pdf]

# Nanosponge-Hydrogel of Octadecyl 3-(3, 5-di-tert-butyl-4-hydroxyphenyl) propanoate of *Alcaligenes faecalis*

Sayed E. El-Sayed[1], Neveen A. Abdelaziz[1,] Ghadir S. El-Housseiny[2], Khaled M. Aboshanab[2]*

[1]Department of Microbiology and Immunology, Faculty of Pharmacy, Ahram Canadian University, Sixth of October City, Giza 12451, Egypt

[2]Department of Microbiology and Immunology, Faculty of Pharmacy, Ain Shams University, Organization of African Unity St., Abbassia, Cairo 11566, Egypt

*** Corresponding Author: Khaled M. Aboshanab**

Address: Department of Microbiology and Immunology, Faculty of Pharmacy, Ain Shams University, Organization of African Unity St., Abbassia, Cairo 11566, Egypt.

E-mail: aboshanab2012@pharma.asu.edu.eg

Mobile: +201-0075-82620

Fax: (202)24051107

https://orchid.org/0000-0002-7608-850X

**Authors' e-mails**

Sayed E. El-Sayed: sayed.emad@acu.edu.eg

Khaled M. Aboshanab: aboshanab2012@pharma.asu.edu.eg

Neveen A. Abdelaziz: neveen.abdelaziz@acu.edu.eg

Ghadir S. El-Housseiny: ghadir.elhossaieny@pharma.asu.edu.eg

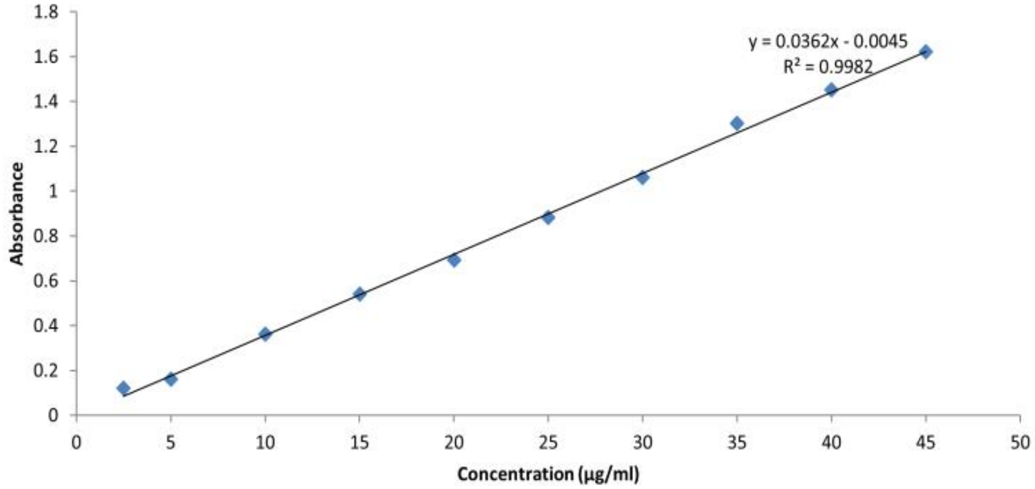


**Fig.S1** Standard curve of ODHP

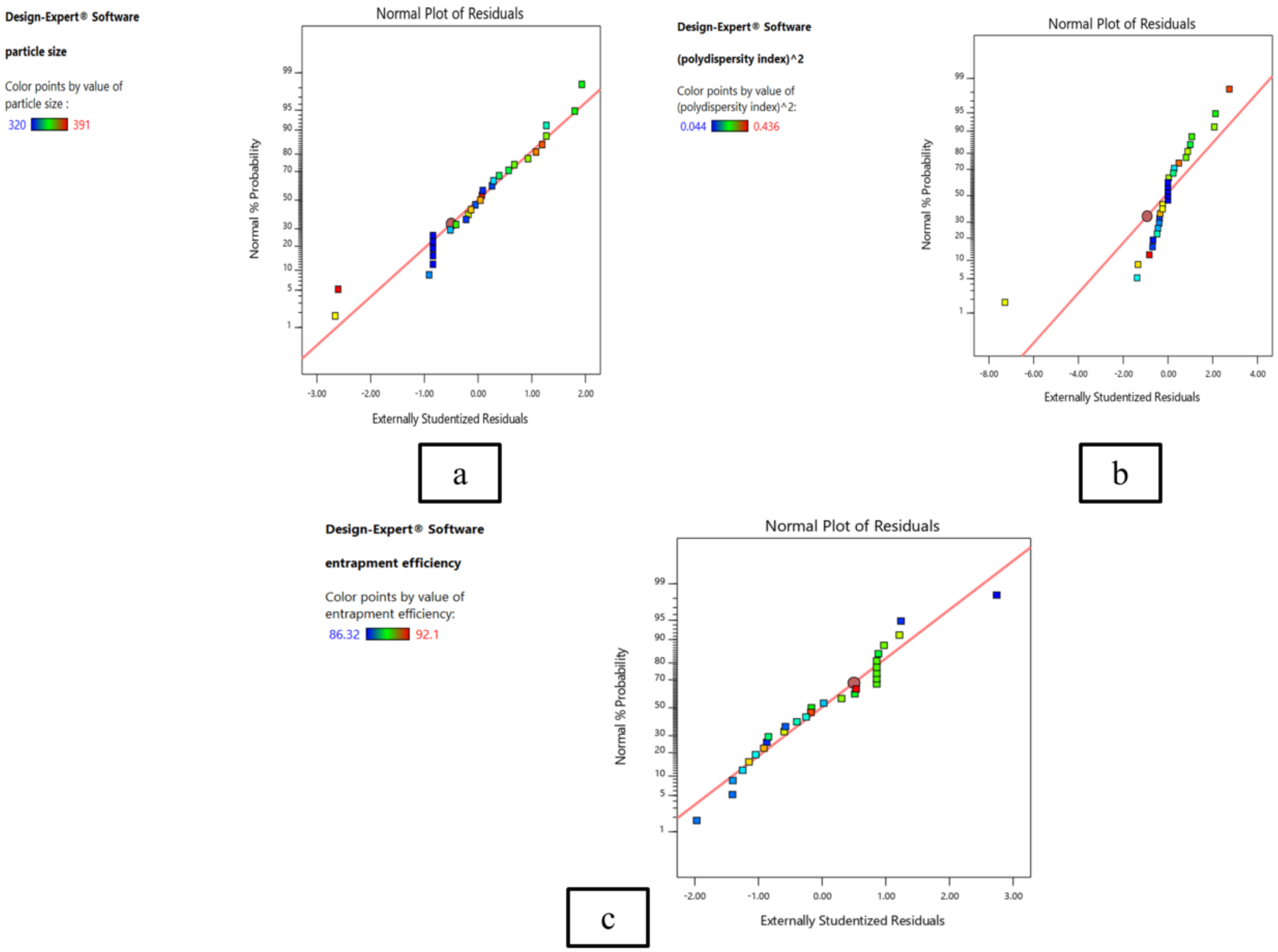


**Fig.S2** The normal probability plots of residuals for (a) PS, (b) PDI, and (c) EE%

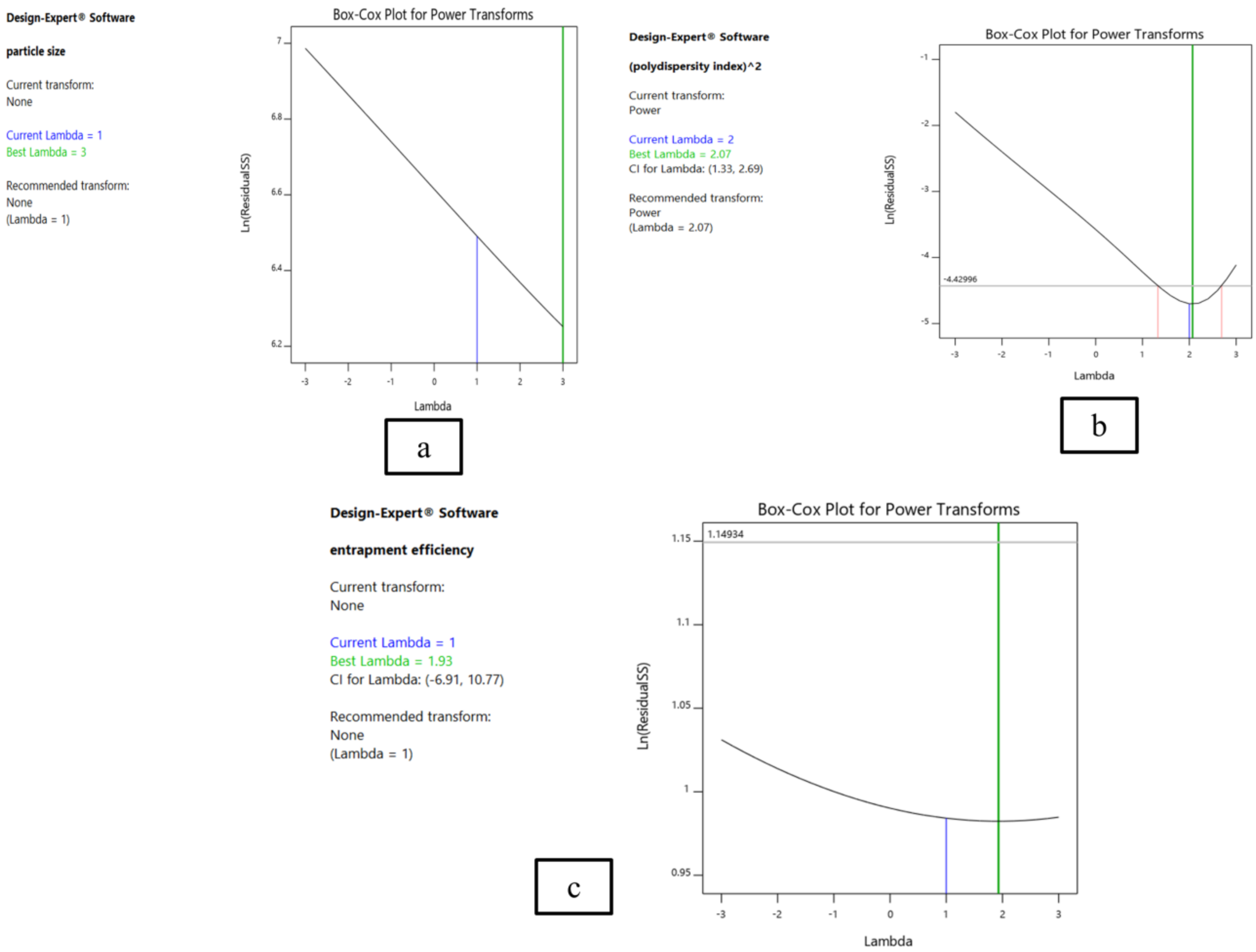


**Fig.S3** The Box Cox plots of (a) PS, (b) PDI, and (c) EE%

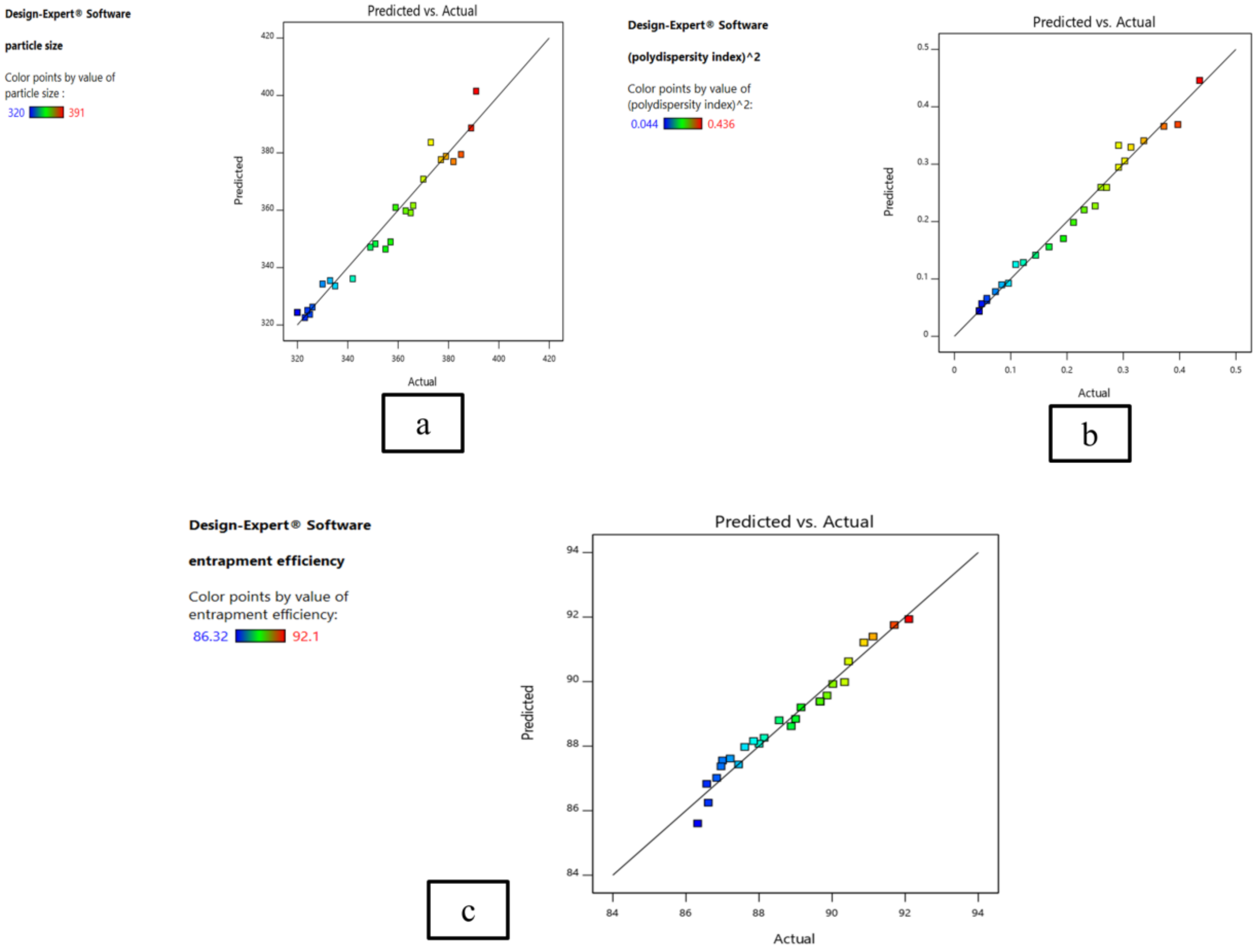


**Fig.S4** The predicted versus actual values plot of (a) PS, (b) PDI, and (c) EE%

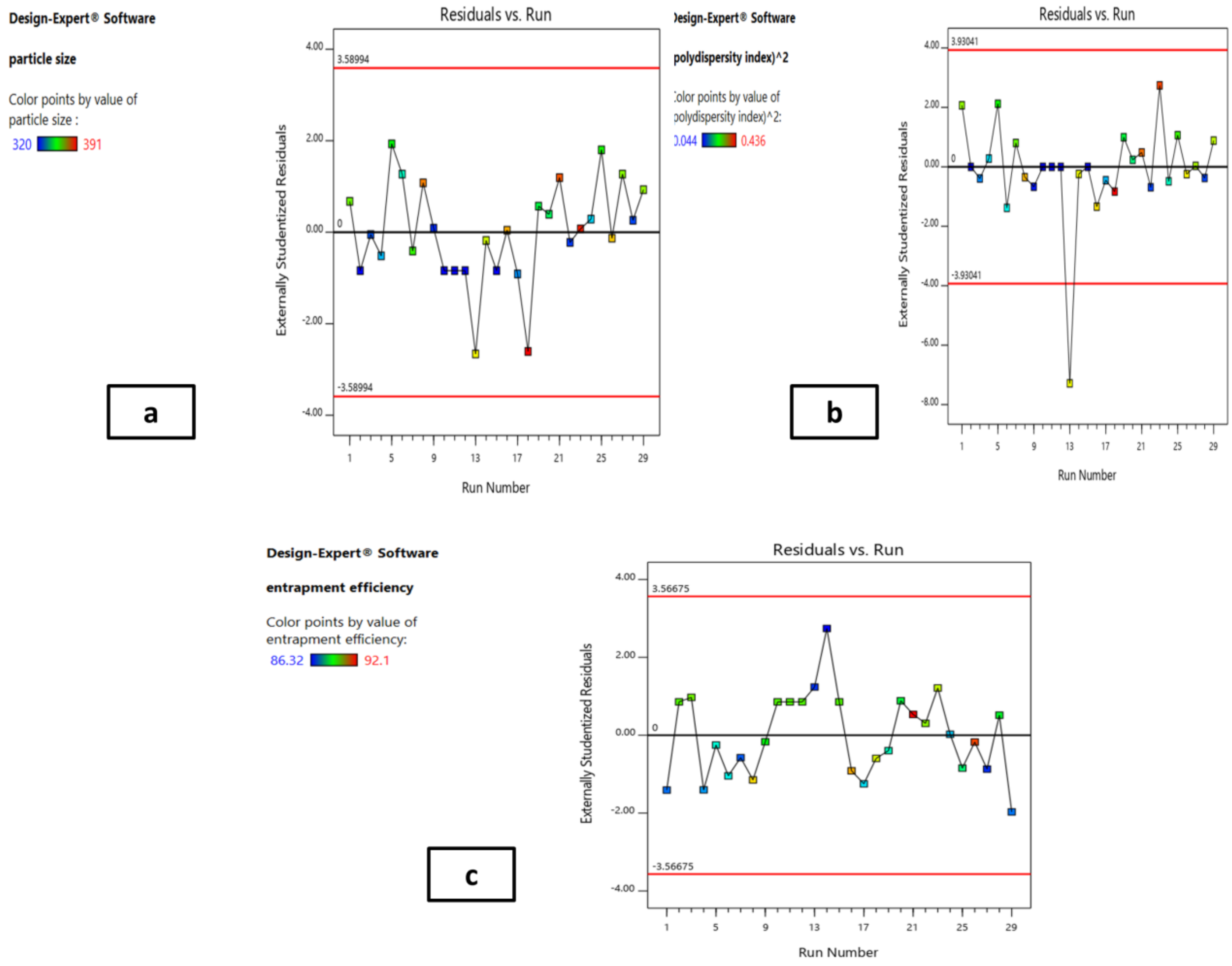


**Fig.S5** The residuals versus Run number plot of (a) PS, (b) PDI, and (c) EE%

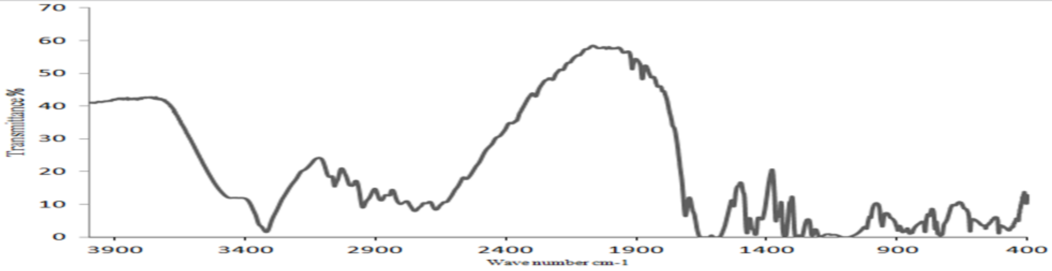
70
60
50
40
30
20
10
0
Transmittance %
3900
3400
2900
2400
1900
1400
900
400
Wave number cm-1

(a)

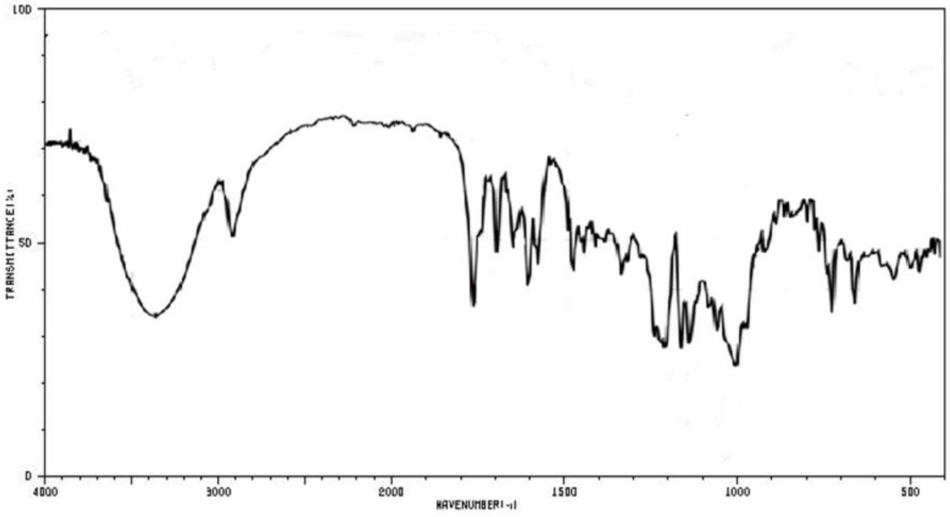
100
50
0
TRANSMITTANCE[%]
4000
3000
2000
1500
1000
500
WAVENUMBER[-1]

(b)

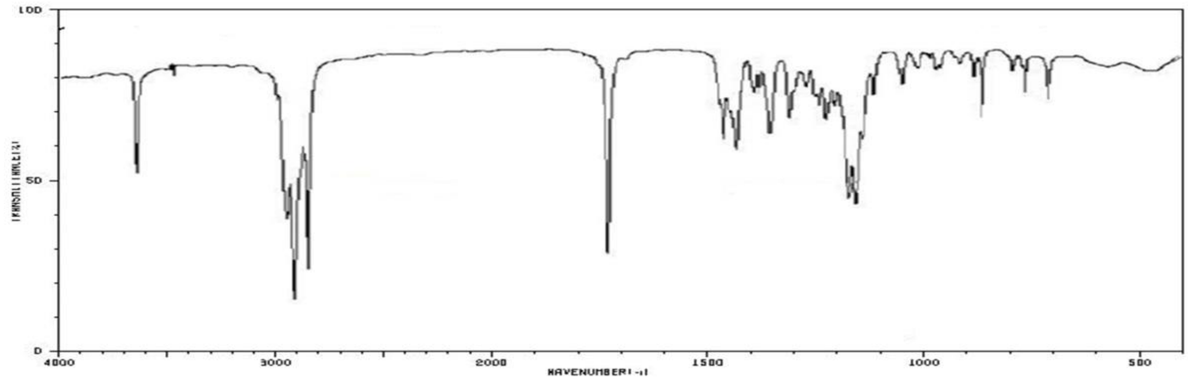

(c)

**Fig.S6** FTIR spectra of (a) ODHP-NS complex (b) β-CD and (c) ODHP

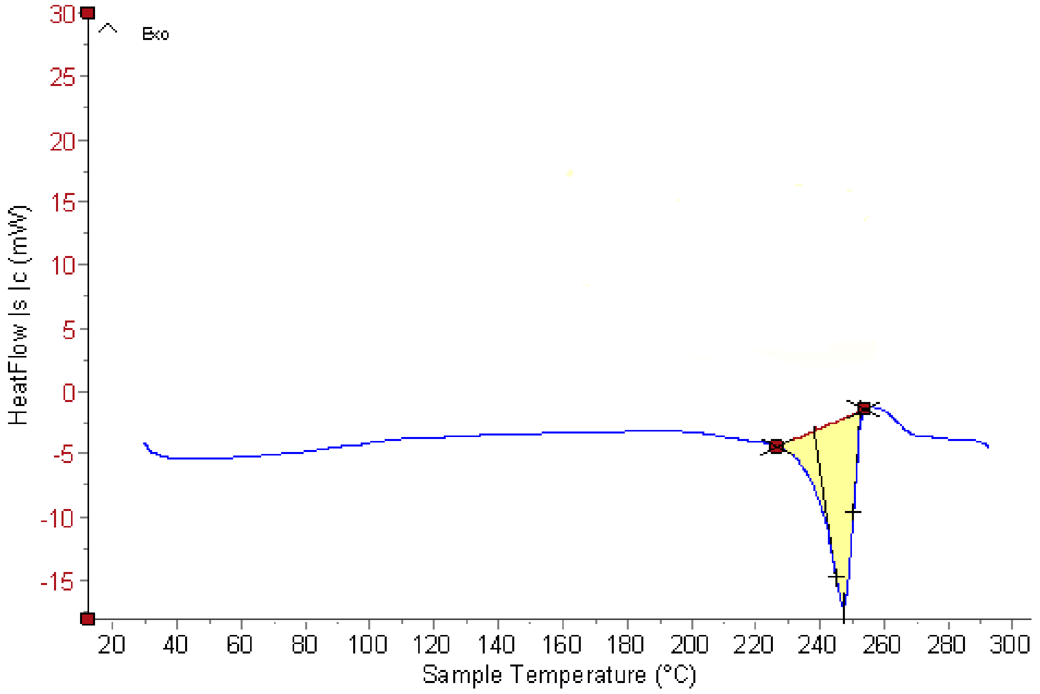


(a)

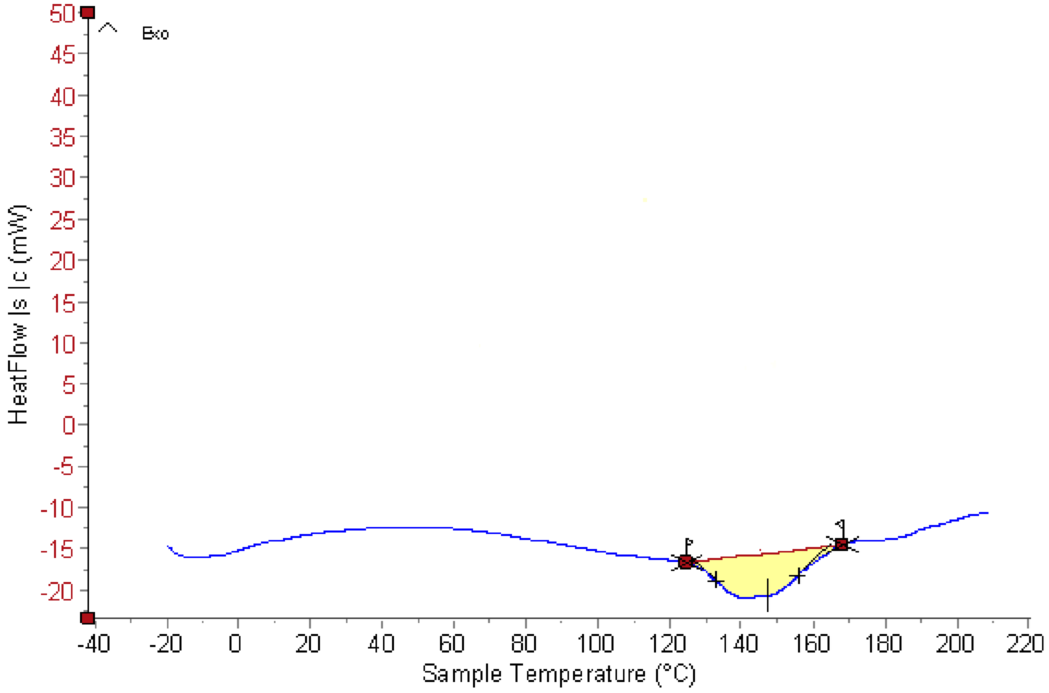


(b)

**Fig.S7** Differential scanning calorimetry thermograms of (a) pure ODHP (b) optimized ODHP-loaded nanosponge formulation

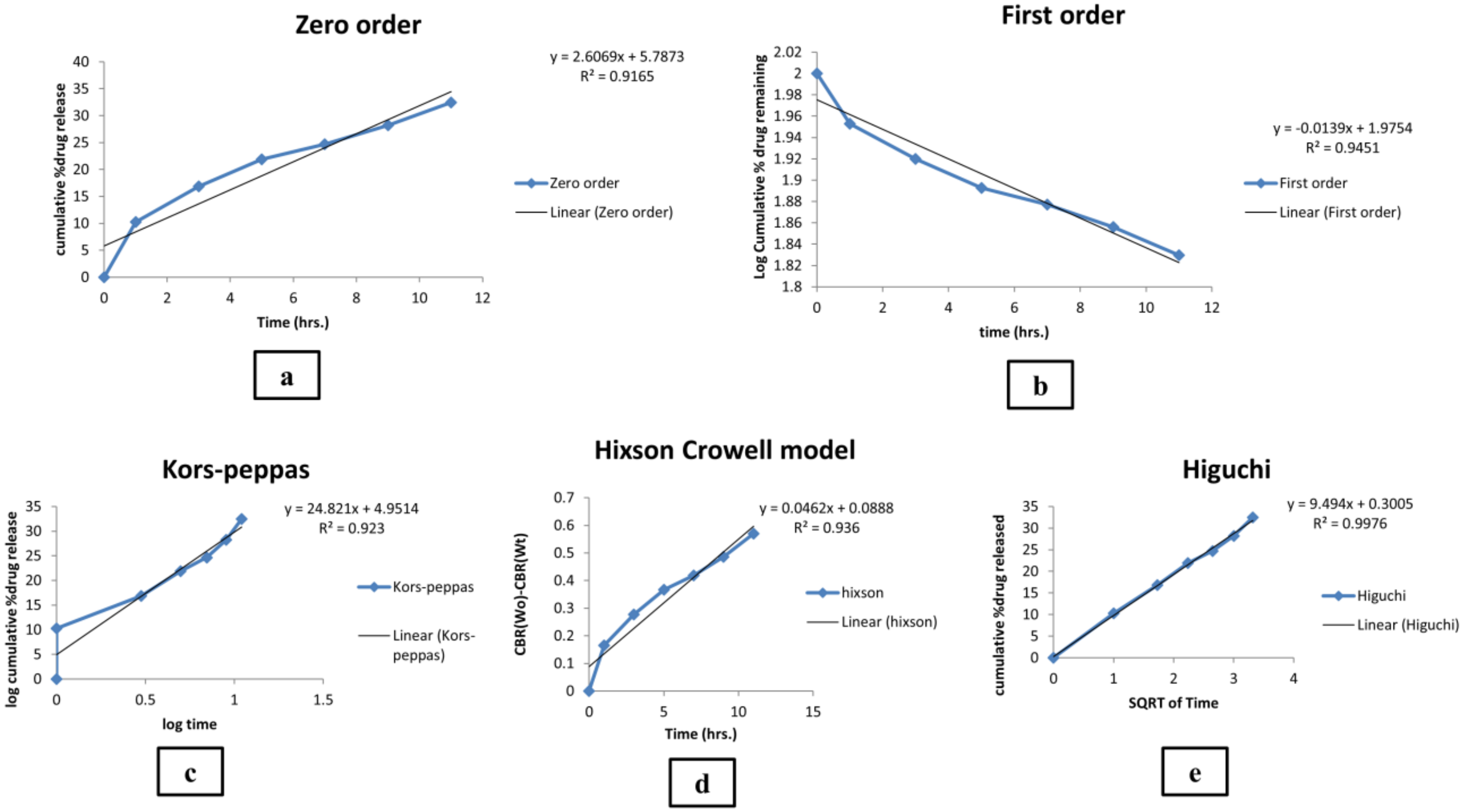


**Fig.S8** Various kinetic models of the in-vitro release data **(a)** Zero order, **(b)** First order, **(c)** Korsmeyer and Peppas, **(d)** Hixson and Crowell model and **(e)** Higuchi ' s model

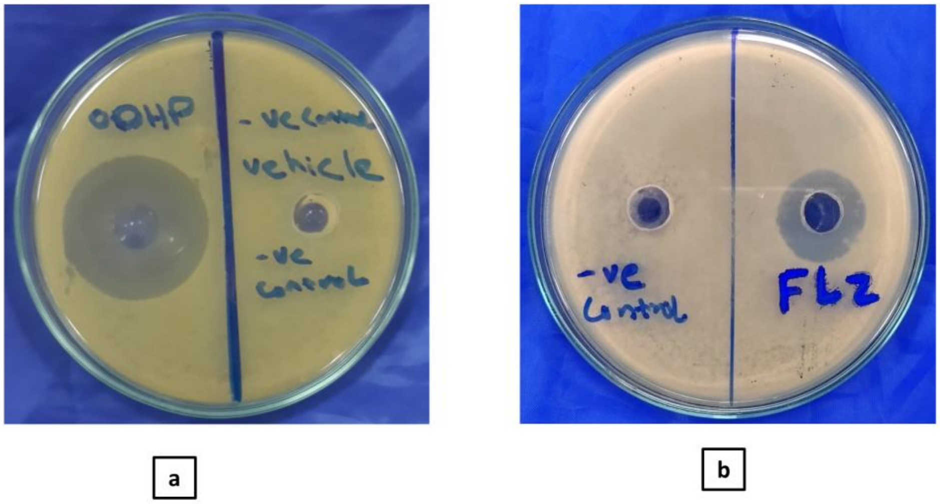


**Fig.S9** In vitro antifungal activity of (a) ODHP-NS-HG (b) Fluconazole (FLZ) against *Candida albicans* ATCC10231

**Table S1** Evaluation of different gel formers for the preparation of hydrogel

| Visual characteristics | Sodium alginate | CMC | Poloxamer 188 | HPMC E4 | Carbopol 940 |
|---|---|---|---|---|---|
| Grittiness | Yes (++++) | NO | Yes (+++) | Yes (++) | NO |
| Stickiness | Yes (++++) | NO | Yes (++) | Yes (++) | NO |
| Spreadability | Very low (++++) | VERY GOOD | Fairly good | Low (++) | EXCELLENT |
| Formation of clumps | Yes (+++++) | Low | NO | NO | NO |

**Table S2** Different gel compositions of carbopol 940

| Component | Formulation code | | | | | |
|---|---|---|---|---|---|---|
| | HG-1 | HG-2 | HG-3 | HG-4 | HG-5 | HG-6 |
| Carbopol 940 (w/v %) | 0.2 | 0.5 | 0.8 | 1 | 1.2 | 1.5 |
| Propylene glycol: distilled water ratio (ml) | 30:70 | 30:70 | 30:70 | 30:70 | 30:70 | 30:70 |
| Triethanolamine (ml) | 2 | 2 | 2 | 2 | 2 | 2 |
| ODHP -NS | 0.15mg | 0.15mg | 0.15mg | 0.15mg | 0.15mg | 0.15mg |
| Methyl paraben (gram) | 1 | 1 | 1 | 1 | 1 | 1 |
| Distilled water (w/w %) (q.s) | 100 | 100 | 100 | 100 | 100 | 100 |

**Table S3** Evaluation of different carbopol 940 hydrogel formulations

| **Visual characteristics** | **HG1** | **HG2** | **HG3** | **HG4** | **HG5** | **HG6** |
|---|---|---|---|---|---|---|
| **Grittiness** | Yes (+++) | NO | Yes (+) | NO | NO | NO |
| **Stickiness** | Yes (+++) | NO | Yes (++) | NO | NO | NO |
| **Spreadability** | Bad | Bad | Fairly good | Good | Good | Good |
| **Homogeneity** | CLUMPS | YES | YES | NO | Yes | NO |
| **Syneresis** | NO | NO | NO | NO | NO | NO |
| **Color** | White | Translucent | Translucent | Translucent | Translucent | Translucent |

**Table S4** Comparison of correlation coefficient ($R^2$) of different kinetic models

| | **Zero order** | **First order** | **Korsmeyer and Peppas** | **Hixson and Crowell model** | **Higuchi ' s model** |
|---|---|---|---|---|---|
| $\mathbf{R^2}$ | 0.9165 | 0.9451 | 0.923 | 0.936 | 0.9976 |

**Table S5** Stability studies data

| Storage conditions | Physical appearance | Mean data ± (SD) | | |
|---|---|---|---|---|
| | | pH | Viscosity (cps) | Drug content |
| **Initial** | Translucent-smooth homogenous | 6.75±0.13 | 1092±4 | 90.96±0.32% |
| **Room temperature** | Translucent -smooth homogenous | 6.92±0.1 | 1060±5 | 86.07±0.16% |
| **5 ± 3 °C** | Translucent -smooth homogenous | 6.80±0.02 | 1185±10 | 89.41±0.58% |
